# Immunotherapy Bridge 2017 and Melanoma Bridge 2017: meeting abstracts

**DOI:** 10.1186/s12967-017-1352-z

**Published:** 2018-01-15

**Authors:** David Carbone, Michael Sharpnack, Kai He, Lisa H. Butterfield, Alexander M. M. Eggermont, Maria Gonzalez-Cao, Niki Karachaliou, Guillermo Crespo, Erika Aldeguer, Ana Drozdowskyj, Ana Giménez-Capitán, Cristina Teixidó, Miguel Angel Molina-Vila, Santiago Viteri Ramírez, Salvador Martin Algarra, Elisabeth Pérez-Ruiz, Iván Márquez-Rodas, Delvys Rodriguez-Abreu, Remei Blanco, Teresa Puértolas, Maria Angeles Royo, Rafael Rosell, Maria Libera Ascierto, Svetlana Hamm, Tanja Wulff, Kerstin Kronthaler, Sabine Schrepfer, Ulrike Parnitzke, Anne Catherine Bretz, Roland Baumgartner, Veronica Ferrucci, Francesco Paolo Pennino, Luisa Dassi, Fatemeh Asadzadeh, Roberto Siciliano, Marianeve Carotenuto, Daniela Spano, Cristina Maria Chiarolla, Adelaide Greco, Monica Cantile, Maurizio Di Bonito, Gerardo Botti, Vandenbussche Jonathan, Gevaert Kris, Massimo Zollo, Teresa Amaral, Ioanna Tampouri, Ulrike Keim, Thomas Eigentler, Claus Garbe, Andrea Forschner, Alessandra Cesano, Sarah Warren, Duane Moogk, Kaitao Li, Zhou Yuan, Shi Zhong, Zhiya Yu, Ivan Liadi, William Rittase, Victoria Fang, Janna Dougherty, Arianne Perez-Garcia, Iman Osman, Navin Varadarajan, Nicholas P. Restifo, Alan Frey, Cheng Zhu, Michelle Krogsgaard, Claire Vanpouille-Box, Silvia C. Formenti, Sandra Demaria, Cristiana Lo Nigro, Alexander Renziehausen, Andreas G. Tzakos, Hexiao Wang, Bhavya Rao, Rubeta Matin, Catherine Harwood, Daniela Vivenza, Federica Tonissi, Marcella Occelli, Lynda Weir, Su Li, Van Ren Sim, Kevin O’Neill, Alan Evans, Alastair Thompson, Peter Szlosarek, Colin Fleming, Charlotte Proby, Nelofer Syed, Marco Merlano, Tim Crook, Robert Ferguson, Danny Simpson, Carlos Martinez, Matjaz Vogelsang, Esther Kazlow, Melissa Wilson, Anna Pavlick, Jeffrey Weber, Ryan Sullivan, Keith Flaherty, Antoni Ribas, Tomas Kirchhoff, Antonio D’Avino, Licia Guida, Augusto Cosacco, Roberta D’Aniello, Piera Maiolino, Teresa Tramontano, Maria R. Sarno, Ida Palazzo, Angela Di Napoli, Gianclaudio Acunzo, Mariarosanna De Fina, Antonia Silvestri, Monica Specchia, Michela Musacchio, Gianfranco Giglio, Francesco Carrozza, Liberato Di Lullo, Gian Marco De Maddi, Adele Venturelli, Elisabetta Gambale, Alessia Gatta, Davide Brocco, Consiglia Carella, Michele De Tursi, Thomas F. Gajewski, Costanza M. Cristiani, Rossana Tallerico, Valeria Ventura, Mariaelena Capone, Gabriele Madonna, Domenico Mallardo, Eliska Selinger, Cinzia Garofalo, Elina Staaf, Ester Simeone, Antonio M. Grimaldi, Genny del Zotto, Elio Gulletta, Gennaro Ciliberto, Alessandro Moretta, Paolo A. Ascierto, Ennio Carbone, Susan Costantini, Angela Sorice, Francesca Capone, Gennaro Ciliberto, Alfredo Budillon, Carolina Cauchi, Grazia Sciancalepore, Cristiana Lo Nigro, Michela Rovera, Chiara Varamo, Zelda Seia, Stefania Palazzini, Fabiana Errico, Davide Basso, Laura Quaranta, Giuseppe Forte, Fulvio Lavagna, Silvia Violante, Paolo Bosio, Laura Lattanzio, Marco C Merlano, Mike Gowen, Jeremy Tchack, Hua Zhou, Keith Giles, Scott Paschke, Una Moran, David Fenyo, Aris Tsirigos, Michael Pacold, Silvana Morello, Claudia Sorrentino, Diana Giannarelli, Aldo Pinto, Federica Fratangelo, Rosaria Falcone, Vito Vanella, Dirk Schadendorf, Karl Lewis, Michele Maio, Lev Demidov, Mario Mandalà, Igor Bondarenko, Christopher Herbert, Andrzej Mackiewicz, Piotr Rutkowski, Alexander Guminski, Grant Goodman, Brian Simmons, Chenglin Ye, Gregory Hooper, Matthew J. Wongchenko, Yibing Yan, Frank Hermann, Astrid Ammendola, Svetlana Hamm, René Bartz, Bulotta Alessandra, Colombo Letizia, Mirabile Aurora, Lazzari Chiara, Mercuri Santo Raffaele, Parolini Danilo, Rizzo Nathalie, Martella Stefano, Modorati Giulio, Brianti Pina, Cestone Enza, Bellinzona Federica, Miserocchi Elisabetta, Gianni Luca, Gregorc Vanesa, Sanjiv Agarwala, B. Mark Smithers, Axel Haushild, Eric Watcher, Luigi Fattore, Ciro Francesco Ruggiero, Domenico Liguoro, Andrea Cerri, Maria Elena Pisanu, Rita Mancini

**Affiliations:** 1 Ohio State University, Columbus, OH USA; 2 Society of ImmunoTherapy of Cancer (SITC), Medicine, Surgery and Immunology and Director, UPCI Immunologic Monitoring and Cellular Products Laboratory, University of Pittsburgh, Pittsburgh, Pennsylvania USA; 3 Cancer Institute Gustave Roussy, Paris, France; 4 Medical Oncology Department, Quirón Salud-Dexeus University Institute, IOR, Barcelona, Spain; 5 Laboratory of Cellular and Molecular Biology, Pangaea Oncology, Quirón-Dexeus University Institute, Barcelona, Spain; 6 IOR-Grupo Quirón Salud-Oncology Department, Hospital Universitari Sagrat Cor, Barcelona, Spain; 7 Hospital Universitario de Burgos, Burgos, Spain; 8 Pivotal, Madrid, Spain; 9 Clínica Universitaria de Navarra, Pamplona, Spain; 10 Hospital Costa del Sol, Oncology Department, REDISSEC, Marbella, Spain; 11 Hospital Gregorio Marañon, Madrid, Spain; 12 Hospital Universitario Insular De Gran Canaria, Las Palmas De Gran Canaria, Spain; 13 Consorcio Sanitario De Terrassa, Terrassa Barcelona, Spain; 14 Aragon’s Health Research Institute, Medical Oncology Department, Miguel Servet University Hospital, Zaragoza, Spain; 15 Hospital Dr Peset, Valencia, Spain; 16 Catalan Institute of Oncology, Germans Trias i Pujol Health Sciences Institute and Hospital, Barcelona, Spain; 17 Medimmune, Gaithersburg, MD USA; 18 The Johns Hopkins University, Baltimore, MD USA; 19 4SC AG, Planegg, 82152 Martinsried, Germany; 20 Dipartimento di Medicina Molecolare e Biotecnologie Mediche, DMMBM, Naples, Italy; 21 CEINGE, Biotecnologie Avanzate, Naples, Italy; 22 European School of Molecular Medicine, SEMM, Milan, Italy; 23 Pathology Unit, Istituto Nazionale Tumori Fondazione “G. Pascale”, Naples, Italy; 24 Department of Medical Protein Research, VIB, Department of Biochemistry, Ghent University, Ghent, Belgium; 25 Center for Dermatooncology, Department of Dermatology, Liebermeisterstr. 25, University Hospital Tuebingen, 72076 Tuebingen, Germany; 26 NanoString Technologies, Inc, Seattle, WA USA; 27 Perlmutter Cancer CenterNew, York University School of Medicine, New York, NY USA; 28 Coulter Department of Biomedical Engineering, Georgia Institute of Technology, Atlanta, GA USA; 29 George W. Woodruff School of Mechanical Engineering, Georgia Institute of Technology, Atlanta, GA USA; 30 Center for Cancer Research, NCI, NIH, Bethesda, MD USA; 31 Department of Chemical and Biomolecular Engineering, University of Houston, Houston, TX USA; 32 Perlmutter Cancer Center, Departments of Cell Biology, New York University, New York, NY USA; 33 Perlmutter Cancer Center, Departments of Dermatology, New York University, New York, NY USA; 34 Perlmutter Cancer Center, Departments of Pathology, New York University, New York, NY USA; 35 Department of Radiation Oncology, Weill Cornell Medicine, New York, NY USA; 36 Department of Oncology, S. Croce & Carle Teaching Hospital, Cuneo, Italy; 37 John Fulcher Neuro-Oncology Laboratory, Division of Brain Sciences, Imperial College London, London, UK; 38 Department of Chemistry, University of Ioannina, Ioannina, Greece; 39 Department of Dermatology, The First Hospital of China Medical University, Shenyang, China; 40 Medical Research Institute, Ninewells Hospital & Medical School, Dundee, UK; 41 Barts and the London School of Medicine and Dentistry, London, UK; 42 Royal Marsden Hospital, Fulham Road, London, UK; 43 Kent Oncology Centre, Maidstone Hospital, Maidstone, UK; 44 Department of Neurosurgery, Charing Cross Hospital, London, UK; 45 Department of Pathology, Ninewells Hospital, Dundee, UK; 46 Breast Surgical Oncology, MD Anderson Cancer Center, Houston, TX USA; 47 Department of Medical Oncology, Bart’s Cancer Centre, London, UK; 48 Department of Dermatology, Ninewells Hospital, Dundee, UK; 49 Department of Oncology, Royal Surrey County Hospital, Guildford, UK; 50 Laura and Issac Perlmutter Cancer Center, New York University School of Medicine, New York City, NY USA; 51 IRCCS Istituto Nazionale Tumori Fondazione “G. Pascale”, Napoli, Italy; 52 S.C. Farmacia, IRCCS Fondazione “G. Pascale”, Napoli, Italy; 53 Department Science of Life, Magna Graecia University, Catanzaro, Italy; 54 U.O.C. Oncologia Medica, Ospedale “A. Cardarelli”, Campobasso, Italy; 55 Pharmacy S.Giovanni, Bosco Hospital A.S.L. Napoli, 1 Centro, Napoli, Italy; 56 Pharmacovigilance Referent A.S.L. Napoli, 1 Centro, Napoli, Italy; 57 Department of Medical, Oral and Biotechnological Sciences, Medical Oncology Unit, “G. D’Annunzio” University, Chieti, Italy; 58 Department of Medicine and Science of Ageing, “G. D’Annunzio” University, Chieti, Italy; 59 Department of Pathology and Department of Medicine (Section of Hematology/Oncology), University of Chicago, Microbiome Center, Chicago, IL USA; 60 Tumor Immunology and Immunopathology Laboratory, Department of Experimental and Clinical Medicine, University “Magna Græcia” of Catanzaro, Campus-Germaneto, Catanzaro, Italy; 61 Department of Microbiology, Cell and Tumorbiology (MTC), Karolinska Institutet, Stockholm, Sweden; 62 Melanoma Cancer Immunotherapy and Innovative Therapy Unit, Istituto Nazionale Tumori Fondazione “G. Pascale”, Naples, Italy; 63 Istituto Giannina Gaslini, Genova, Italy; 64 Department of Health Sciences, University “Magna Græcia” of Catanzaro, Campus-Germaneto, Catanzaro, Italy; 65 Scientific Directorate, IRCCS Istituto Nazionale Tumori Fondazione “G. Pascale”, Naples, Italy; 66 Department of Experimental Medicine and Center of Excellence for Biomedical Research, University of Genoa, Genova, Italy; 67 Experimental Pharmacology Unit, Istituto Nazionale Tumori Fondazione “G. Pascale” - IRCCS, Napoli, Italy; 68 Scientific Directorate, Istituto Nazionale Tumori “Regina Elena”, IRCCS, Roma, Italy; 69 Melanoma Cancer Immunotherapy and Innovative Therapy Unit, Istituto Nazionale Tumori “Fondazione G. Pascale” IRCCS, Napoli, Italy; 70 Oncologia medica-AO Santa Croce E Carle, Cuneo, Italy; 71 Anatomia Patologica-AO Santa Croce E Carle, Cuneo, Italy; 72 CAS–AO Santa Croce E Carle, Cuneo, Italy; 73 LILT, Cuneo, Italy; 74 SS Day Surgery-AO Santa Croce E Carle, Cuneo, Italy; 75 Chirurgia Generale-AO Santa Croce E Carle, Cuneo, Italy; 76 Oncologia – IRCCS, Candiolo, Italy; 77 The Ronald O. Perelman Department of Dermatology, New York University School of Medicine, New York, NY USA; 78 Center for Health Informatics and Bioinformatics, NYU Langone Medical Center, New York, NY USA; 79 CDI Laboratories, Baltimore, MD USA; 80 Department of Radiation Oncology, NYU School of Medicine, New York, NY USA; 81 Division of Hematology & Oncology, Laura and Isaac Perlmutter Cancer Center, New York, NY USA; 82 Department of Pathology, NYU School of Medicine, New York, NY USA; 83 Department of Pharmacy, University of Salerno, Fisciano, Italy; 84 Melanoma, Cancer Immunotherapy and Innovative Therapies O.U, National Cancer Institute “G. Pascale”, Naples, Italy; 85 University of Salerno, Fisciano, Italy; 86 Regina Elena National Cancer Institute, Rome, Italy; 87 S.C. Oncologia Clinica Sperimentale Melanoma Immunoterapia e Terapie Innovative-IRCCS-Fondazione G. Pascale, Naples, Italy; 88 Dipartimento di Farmacia, Università di Salerno, Fisciano, Italy; 89 Istituto Nazionale Tumori Regina Elena, Roma, Italy; 90 Department of Dermatology, University Hospital Essen, Essen, Germany; German Cancer Consortium, Heidelberg, Germany; 91 University of Colorado Comprehensive Cancer Center, Aurora, CO USA; 92 Medical Oncology and Immunotherapy, Center for Immuno-Oncology, University Hospital of Siena, Siena, Italy; 93 N N Blokhin Russian Cancer Research Center, Ministry of Health, Moscow, Russia; 94 Department of Oncology and Haematology, Papa Giovanni XXIII Cancer Center Hospital, Bergamo, Italy; 95 Dnipropetrovsk State Medical Academy, Dnipropetrovsk, Ukraine; 96 Melanoma Unit, Cancer Immunotherapy and Innovative Therapies, Istituto Nazionale Tumori Fondazione “G. Pascale”, Naples, Italy; 97 Bristol Haematology and Oncology Centre, Bristol, UK; 98 Department of Cancer Immunology, Poznan University for Medical Sciences, Med-POLONIA, Poznan, Poland; 99 Department of Soft Tissue/Bone Sarcoma and Melanoma, Maria Sklodowska-Curie Institute – Oncology Center, Warsaw, Poland; 100 Melanoma Translational Research Group, Melanoma Institute Australia, Wollstonecraft, NSW Australia; 101 Genentech, Inc, South San Francisco, CA USA; 102 Roche Products Limited, Welwyn Garden City, UK; 103 Department of Oncology, Division of Experimental Medicine, San Raffaele Scientific Institute, Milan, Italy; 104 Dermatology and Cosmetology Unit-San Raffaele Scientific Institute, Milan, Italy; 105 Division of Plastic and Reconstructive Surgery, San Raffaele Scientific Institute, Milan, Italy; 106 Unit of Gastrointestinal Surgery, San Raffaele Scientific Institute, Milan, Italy; 107 Department of Pathology, San Raffaele Scientific Institute, Milan, Italy; 108 Department of Ophthalmology, San Raffaele Scientific Institute, Milano, Italy; 109 St Luke’s University Hospital and Health Network, Easton, Pennsylvania USA; 110 Princess Alexandra Hospital, Brisbane, Queensland Australia; 111 Department of Dermatology, University Hospital Schleswig-Holstein, Kiel, Germany; 112 Provectus Biopharmaceuticals, Inc, Knoxville, Tennessee United States; 113 Istituto Nazionale per lo Studio e la Cura dei Tumori “Fondazione G. Pascale”, Naples, Italy; 114 Dipartimento di Medicina Sperimentale e Clinica, Università degli Studi di Catanzaro “Magna Graecia”, Catanzaro, Italy; 115 Dipartimento di Medicina Clinica e Molecolare, Sapienza Università di Roma, Rome, Italy

## Immunotherapy bridge 2017—Keynote speaker presentations

### System biology session: immunology

#### K1 Genomics and immunotherapy in lung cancer: tumor mutation burden, mutations affecting antigen presentation, immune recognition, and genome integrity

##### David Carbone, Michael Sharpnack, Kai He

###### Ohio State University, Columbus, OH, USA

*Journal of Translational Medicine 2018*, **16 (Suppl 1):**K1

Immunotherapy approaches targeting the PD-1 pathway have shown some clinical benefits in a fraction of patients with lung cancer, but expression of PD-L1 has proven to be an imperfect biomarker of efficacy. Recent studies have shown that tumor mutation burden (TMB) is also correlated with outcome and that it appears to be independent of PD-L1. TMB, however, only indirectly measures the number of neoantigenic peptides presented on tumor cell surface class I MHC and predicted MHC matches may be an even better predictor of benefit. In addition, mutations in genes such as LKB1 may modulate the immune response, and mutations in the antigen presentation pathway may block it altogether. Mutations in DNA repair pathway genes may increase the number of potential neoantigens. Thus in depth analysis of tumor somatic genomics could lead to better patient selection for immunotherapy.

### SITC session—evolving topics in cancer immunotherapy

#### K2 Discussion about evolving topics in cancer immunotherapy

##### Lisa H. Butterfield

###### Medicine, Surgery, Immunology and Clinical and Translational Science, University of Pittsburgh, UPMC Hillman Cancer Center Research Pavilion, Room 1.27, Pittsburgh, Pennsylvania, USA

*Journal of Translational Medicine 2018*, **16 (Suppl 1):**K2

Dendritic cell vaccines have been used to promote anti-tumor immune responses in over 200 clinical trials. While clinical responses have been limited, DC could play a critical role in promoting T cell infiltration into tumors which could be capitalized on with checkpoint blockade. Combination approaches including DC could also lead to improved outcomes. We tested an antigen-engineered DC vaccine with or without subsequent IFNα in melanoma. Trial results and immune biomarker data will be presented.

### Immunotherapy in oncology: data from clinical trials

#### K3 Consequences of rapid evolution of adjuvant therapy landscape in melanoma

##### Alexander M. M. Eggermont

###### Cancer Institute Gustave Roussy, Paris, France

*Journal of Translational Medicine 2018*, **16 (Suppl 1):**K3

The spectacular outcomes of the phase III trials regarding nivolumab versus ipilimumab in fully resected stage IIIB/C-IV and of the combination of dabrafenib (D) plus trametenib (T) in BRAF mutant stage III patients demonstrate that effective treatments in advanced melanoma are also highly effective in the adjuvant setting. In 2016 an overall survival benefit with adjuvant high dose ipilimumab was demonstrated, and the EORTC1325 trial comparing pembrolizumab versus placebo will complete the picture early 2018. Toxicity profiles are in line with the experience in advanced melanoma, i.e. favorable for the anti-PD1 agents and for D+T, and problematic for ipilimumab. The 2017 outcomes are practice changing and put an end to the use of interferon and ipilimumab. In countries with only access to interferon, its use can be restricted to patients with ulcerated melanoma, based on the individual patients data meta-analysis recently published. Because of the results of the MSLT2 trial, completion lymph node dissection (CLND) will decrease sharply leading to a lack of optimal prognostic information. Prognosis in SN-positive stage IIIA/B patients is extremely heterogeneous with 5 year survival rates varying from 90% to 40%, and depends mostly on number of positive nodes identified by CLND. This information is crucial for clinical decision making. How to guarantee optimal staging information needs to be discussed urgently. Further improvements of adjuvant therapies will have to address all these questions as well as the exploration of neo-adjuvant use of active drugs and combination approaches. Important paradigm shifts in the management of high risk melanoma patients are upon us.

## Immunotherapy bridge 2017—oral presentations

### System biology session: immunology

#### O1 Interferon-gamma (INFG), a predictive factor of response to immune checkpoint blockade (ICB) in melanoma and non-small cell lung cancer (NSCLC)

##### Maria Gonzalez-Cao^1,2^, Niki Karachaliou^2,3^, Guillermo Crespo^4^, Erika Aldeguer^2^, Ana Drozdowskyj^5^, Ana Giménez-Capitán^2^, Cristina Teixidó^2^, Miguel Angel Molina-Vila^2^, Santiago Viteri Ramírez^1,2^, Salvador Martin Algarra^6^, Elisabeth Pérez-Ruiz^7^, Iván Márquez-Rodas^8^, Delvys Rodriguez-Abreu^9^, Remei Blanco^10^, Teresa Puértolas^11^, Maria Angeles Royo^12^, Rafael Rosell^1,2,3,13^, on behalf of the Spanish Melanoma Group (GEM)

###### ^1^Quirón Salud-Dexeus University Institute, IOR, Medical Oncology Department, Barcelona, Spain; ^2^Pangaea Oncology, Quirón-Dexeus University Institute, Laboratory of Cellular and Molecular Biology, Barcelona, Spain; ^3^Hospital Universitari Sagrat Cor, IOR-Grupo Quirón Salud-Oncology Department, Barcelona, Spain; ^4^Hospital Universitario de Burgos, Burgos, Spain; ^5^Pivotal, Madrid, Spain; ^6^Clínica Universitaria de Navarra, Pamplona, Spain; ^7^Hospital Costa del Sol, Oncology Department, REDISSEC, Marbella, Spain; ^8^Hospital Gregorio Marañon, Madrid, Spain; ^9^Hospital Universitario Insular De Gran Canaria, Las Palmas De Gran Canaria, Spain; ^10^Consorcio Sanitario De Terrassa, Terrassa Barcelona, Spain; ^11^Miguel Servet University Hospital, Aragon’s Health Research Institute, Medical Oncology Department, Zaragoza, Spain; ^12^Hospital Dr Peset, Valencia, Spain; ^13^Catalan Institute of Oncology, Germans Trias i Pujol Health Sciences Institute and Hospital, Barcelona, Spain

*Journal of Translational Medicine 2018*, **16 (Suppl 1):**O1

**Background:** PD-L1 is up-regulated via INFG in a STAT1- and NFκB-dependent manner. We explored whether INFG expression in pre-treatment tumors is associated with the activity of ICB in NSCLC and melanoma patients. The role of inflammation-associated transcription factors STAT3, IKBKE and STAT1 was also examined.

**Methods:** Total RNA from 17 NSCLC and 21 melanoma patients, was analyzed by qRT-PCR. STAT3 and Rantes, YAP1 and CXCL5, DNMT1, RIG1 and TET1, EOMES, INFG (encoding for INFγ), PD-L1 and CTLA4, IKBKE and NFATC1 mRNA were examined. PD-L1 protein expression in tumor and immune cells and stromal infiltration of CD8+ T cells were also evaluated.

**Results:** 17 previously treated NSCLC patients received nivolumab; 71% lung adenocarcinoma, 71% male, 53% smokers, 35% KRAS mutant, 88% EGFR wild-type (wt). 21 previously treated melanoma patients received pembrolizumab; 67% male, 67% BRAF wt. PFS to nivolumab was significantly longer in NSCLC patients with high vs low INFG expression (5.12 vs 2 months, p = 0.0124). PFS to pembrolizumab was significantly longer in melanoma patients with high vs low INFG expression (4.99 vs 1.86 months, p = 0.0099). Significantly longer OS was observed for melanoma patients with high vs low INFG expression (not reached vs 3.10 months, p = 0.0183). There was a trend for longer OS for NSCLC patients with high vs low INFG expression (10.15 vs 4.86 months, p = 0.0687). A survival plateau was only observed for patients with high INFG levels: OS_21m_ 60% vs 0% for melanoma, and 15% versus 0% for NSCLC). Clinical benefit (CB)(objective response or stable disease) was observed in 71.43% of NSCLC patients with high INFG levels versus 0% in patients with low levels; for melanoma patients CB was observed in 71.4% of patients with high IFNG levels versus 20% for patients with low INFG. The in tumor and immune cells did not affect the outcome to ICB. IKBKE was positively correlated with INFG and PD-L1 expression (NSCLC Spearman’s ρ = 0.58 and 0.65; melanoma Spearman’s ρ = 0.61 and 0.59), and STAT3 expression was loosely anticorrelated with PD-L1 expression (NSCLC Spearman’s ρ = − 0.21; melanoma Pearson’s ρ = − 0.01). The rest of the biomarkers explored did not affect the outcome to immunotherapy.

**Conclusions:** NSCLC and melanoma patients with intermediate/high INFG mRNA expression exhibited longer PFS and OS and higher disease control rates with anti PD-1 therapies even when the levels of PD-L1 expression were low.

#### O2 Biomarkers for cancer immunotherapy: predicting the immune resistance through gene expression profile

##### Maria Libera Ascierto^1,2^

###### ^1^Medimmune, Gaithersburg, MD, USA; ^**2**^The Johns Hopkins University, Baltimore, MD, USA

*Journal of Translational Medicine 2018*, **16 (Suppl 1):**O2

**Background:** Surgery, radiation and chemotherapy have been long considered the three pillars of cancer care. A fourth pillar, immunotherapy, recently expanded providing an exciting new treatment option for many patients. In this regard, monoclonal antibodies blocking PD-1/PD-L1 immune checkpoint have shown promising clinical results [1].

Despite these very encouraging results, the majority of patients do not respond to immunotherapy regimens as monotherapy, leading to an urgency to identify biomarkers that accurately predict which patients will benefit from treatment and potentially actionable mechanisms of resistance in order to set successful combinatorial approaches [2]. To this end, gene-expression profiling has been here applied on lesions derived from patients with renal cell carcinoma (RCC), melanoma and classical Hodgkin lymphoma (CHL) thus unveiling novel and paradoxical relations between cancer and immune system leading to immunotherapy resistance.

**Method:** RNA was isolated from (i): 13 formalin-fixed, paraffin-embedded (FFPE) pre-PD-1 treatment tumor biopsies derived from RCC patients; (ii): 10 FFPEs regressing/progressing cutaneous metastases derived from on autopsy case of melanoma; (iii): 24 FFPEs derived from CHL Epstein Barr virus (EBV) positive (+) and negative (−) patients.

RNA was subjected to whole genome microarray and multiplex quantitative (q) RT-PCR.

**Results:** In renal cell carcinoma, gene expression profile highlighted metabolic and immunologic molecules to be associated with the effective response to immunotherapy with anti- PD-1 blockade [3]. In melanoma, transcriptional signatures mostly associated with epithelial to mesenchymal transition (EMT) and accumulation of neutrophils were found to be associated with PD-1 blockade therapy resistance [4]. In CHLs, results revealed a dichotomous cellular and cytokine immune milieu in EBV+ vs EBV− CHL [5]. Particularly, EBV+ tumors displayed a T helper 1 (Th1) profile while EBV-tumors manifested a pathogenic Th17 profile and ongoing engagement of the interleukin-23 (IL-23)/IL-17 axis^5^. These findings suggest that drugs blocking the IL-23/IL-17 axis, may enhance the therapeutic impact of immunotherapy in EBV–CHL.

**Conclusions:** Many pathways might determine the clinical response to immunotherapies in cancer patients, thus suggesting that in the evaluation of biomarkers associated with response to immunotherapy, all intersections between immunological, genetic and tissue specific factors must be evaluated. Merging together the usage of high-throughput screenings, bioinformatic analysis and immune biology assays might be necessary to establish a framework for describing the diversity of these interactions with the aim to focus on features that help guide immunotherapeutic treatment choices on an individual basis (i.e. personalized medicine).

**Acknowledgments** Studies here described have been conducted at Johns Hopkins University during author’s post-doctoral fellowship. Special thanks goes to S.L. Topalian for post-doctoral mentorship, insight suggestions and continuous support. The author also thanks E.J. Lipson, J.M. Taube, T.L. McMiller, A.E. Berger, A.S. Duffield, C.G. Drake, R.F. Ambinder, and D.M. Pardoll for advices, technical assistance and data analysis.

**References**

1. Ascierto ML, Melero I, Ascierto PA. Melanoma: from incurable beast to a curable bet. The success of immunotherapy. Front Oncol. 2015;5:152.
2. Bedognetti D, Marincola FM, Wang E, Ascierto ML. Molecular profiling of immunotherapeutic resistance. In: Prendergast GC, Jaffee EM. Cancer immunotherapy: immune suppression and tumor growth. 2nd ed. Elsevier; 2013.
3. Ascierto ML, McMiller TL, Berger AE, Danilova L, Anders RA, Netto GJ, Xu H, Pritchard TS, Fan J, Cheadle C, Cope L, Drake CG, Pardoll DM, Taube JM, Topalian SL. The intratumoral balance between metabolic and immunologic gene expression is associated with anti-PD-1 response in patients with renal cell carcinoma. Cancer Immunol Res. 2016;4:726–733.
4. Ascierto ML, Makohon-Moore A, Lipson EJ, Taube JM, McMiller TL, Berger AE, Fan J, Kaunitz GJ, Cottrell TR, Kohutek ZA, Favorov A, Makarov V, Riaz N, Chan TA, Cope L, Hruban RH, Pardoll DM, Taylor BS, Solit DB, Iacobuzio-Donahue CA, Topalian SL. Transcriptional mechanisms of resistance to anti-PD-1 therapy. Clin Cancer Res. 2017;23(12):3168–3180.
5. Duffield AS, Ascierto ML, Anders RA, Taube JM, Meeker AK et al. Chen S, McMiller TL, Phillips NA, Berger AE, Pardoll DM, Topalian SL, Ambinder RF. Th17 immune microenvironment in Epstein–Barr virus negative Hodgkin lymphoma: implications for immunotherapy. Blood Adv **(In press)**.

#### O3 4SC-202 induces inflamed tumor microenvironment, strongly enhances tumor infiltration with cytotoxic T cells and primes tumors for anti-PD-1/PD-L1 therapy

##### Svetlana Hamm, Tanja Wulff, Kerstin Kronthaler, Sabine Schrepfer, Ulrike Parnitzke, Anne Catherine Bretz, Roland Baumgartner

###### 4SC AG, 82152 Planegg, Martinsried, Germany

*Journal of Translational Medicine 2018*, **16 (Suppl 1):**O3

**Background:** Various HDAC inhibitors were described as beneficially affecting anti-tumoral immune response. Although different HDAC inhibitors were investigated in syngeneic tumor models, the mode of anti-tumoral action is not yet fully understood. Here, we analyzed the anti-tumoral mode-of-action (MOA) of 4SC-202, an orally available clinical stage epigenetic small molecule inhibitor targeting histone deacetylases (HDAC) class I. To ensure that the conclusions would be relevant for the clinical situation we used a clinically equivalent dosage regimen.

**Materials and methods:** Anti-tumoral efficacy and the impact on tumor microenvironment (TME) were analyzed in syngeneic colorectal CT26 and C38 models in immunocompetent BALB/c or in nude/irradiated BALB/c mice. A broad spectrum HDAC inhibitor was used for comparison. Transcriptome analysis was performed by RNA-Seq, and the composition of immune cell subpopulations was determined by flow cytometry.

**Results:** 4SC-202 treatment significantly inhibited growth of CT26 and C38 tumors. A competent immune system was apparently necessary for the anti-tumoral effect of 4SC-202 since its tumor-reducing effect was lost in immunocompromised mice. 4SC-202 treatment increased IFN-γ and chemokine expression, and reduced pro-inflammatory IL-1 and IL-23 in the TME of CT26 tumors. Detailed analysis revealed that 4SC-202 increased the number of cytotoxic CD8+ T cells (CTLs) in TME of both, T cell-inflamed C38 as well as of non-T-cell-inflamed CT26 tumors without affecting the number of CTLs in blood. In contrast, a broad-spectrum HDAC inhibitor tested in the same model demonstrated anti-tumoral efficacy but did not affect the number of CTLs in tumors demonstrating that HDAC inhibitors employ different MOAs for their anti-tumoral response and that the effect on CTLs is not attributed to HDAC inhibition in general. Since the T cell abundance is pre-requisite for the efficacy of PD1/PD-L1 blockade, combinations of 4SC-202 with anti-PD-1/anti-PD-L1 antibodies were tested in C38 and CT26 models, respectively. The combined treatment was more efficacious than monotherapies and resulted in significantly longer survival in both models with 55% tumor-free animals in C38 model.

**Conclusions:** 4SC-202 already demonstrated a favorable safety profile in a phase I clinical trial with relapsed or refractory hematological malignancies with two objective responses (1 CR, 1 PR) and disease stabilizations in several patients. 4SC-202’s immune priming capacity offers further options for clinical development of 4SC-202 in combination with various cancer immunotherapy approaches. Combination of 4SC-202 with PD-1 blockade will be evaluated in a Phase Ib/II clinical study in advanced cutaneous melanoma patients refractory/non-responding to treatment with anti-PD-1 antibodies (‘SENSITIZE’, NCT03278665).

### Immunotherapy beyond melanoma

#### O4 A new triple negative breast cancer (TNBC) murine model for in vivo preclinical immunotherapies

##### Veronica Ferrucci^1,2,3^, Francesco Paolo Pennino^1,2^, Luisa Dassi^2^, Fatemeh Asadzadeh^2^, Roberto Siciliano^2^, Marianeve Carotenuto^1,2^, Daniela Spano^1,2^, Cristina Maria Chiarolla^1,2^, Adelaide Greco^1,2^, Monica Cantile^4^, Maurizio Di Bonito^4^, Gerardo Botti^4^, Vandenbussche Jonathan^5^, Gevaert Kris^5^, Massimo Zollo^1,2,3^

###### ^1^Dipartimento di Medicina Molecolare e Biotecnologie Mediche, DMMBM, Naples, Italy; ^2^CEINGE, Biotecnologie Avanzate, Naples, Italy; ^3^European School of Molecular Medicine, SEMM, Milan, Italy; ^4^Pathology Unit, Istituto Nazionale Tumori Fondazione “G. Pascale”, Naples, Italy; ^5^Department of Medical Protein Research, VIB, Department of Biochemistry, Ghent University, Ghent, Belgium

*Journal of Translational Medicine 2018*, **16 (Suppl 1):**O4

**Background:** Triple Negative Breast Cancers (TNBCs), lacking hormone receptors and HER2, are highly metastatic and chemoresistant and metastatic events (mBC) are the most common cause of death in women. Tumour microenvironment (TME) is a complex network of cells that supports tumorigenesis and metastatic spread. Among the immune cells of TME, Tumour-Associated-Macrophages (TAMs) are the most abundant in BC by regulating invasion, metastases and chemoresistance. TAMs can acquire distinct phenotypes in response to different signals. M2-polarized-TAMs have immunosuppressive activities by expressing inflammatory molecules.

Prune-1 belongs to DHH (Asp-His-His) phosphoesterase superfamily with an exopolyphosphatase activity [2, 8]. The overexpression of Prune-1 is correlated with metastases and poor prognosis in several tumours including BC [8]. Prune-1 has been also found to induce Epithelial-Mesenchymal-Transition (EMT) and metastatic dissemination through the enhancement of canonical TGF-β signalling by counterbalancing its inhibition operated by NM23-H1 [3, 4]. Furthermore, we also have evidences that lung cancer progression is driven by Prune-1 via canonical WNT signalling in autocrine and paracrine manner via Wnt3a secretion [1].

**Results:** We identified Prune-1 with the ability to recruit and polarize TAMs toward a pro-tumorigenic M2-phenotype within the TME of TNBC using a double Genetically Engineered Mouse (GEM) model of TNBC over-expressing both Prune-1 and Wnt-1 in mammary glands (MMTV-Prune1/Wnt1; generated through the use of vectors construct containing the human transgene cDNAs under the control of Mouse Mammary Tumour Virus [MMTV] promoter). These novel Genetically Engineered Mouse (GEMs) model of TNBC (MMTV-Prune1-WNT1) develop BCs with 100% penetrance between months 2–3 of life (starting after the mammary gland is fully developed) and importantly they always generate lung metastases, while the single transgenic MMTV-Wnt1 TNBC models [5, 7] is not able to make them. These GEMs were then crossed with the receptor 2 of VEGF (VEGFR2) promoter driving firefly luciferase gene expression. Results are indicating that once these recombinant animals MMTV-Wnt1 or MMTV-Prune1/Wnt1 develop tumours activating VEGF then these initiating tumorigenic cells can be visible by in vivo bioluminescence imaging (BLI) luciferase technology. Studies in primary cells derived from the BC generated by these GEM models, indicate that the over-expression of Prune-1 is responsible for the activation of intracellular pathways (i.e. TGF-β, FAK and NF-κB) and for both the activation and polarization of macrophages in vitro shown by the activation of JAK-STAT3 and NF-κB signalling cascades and the increase of inflammatory cytokines (e.g. Arg1, iNOS, MMP9 and IL1β in those macrophages treated with conditioned media derived from MMTV-Prune-1/Wnt-1 primary cells. This thus confirms Prune-1 able to polarize TAMs toward an M2-phenotype.

**Conclusions:** We generate a TNBC murine model with lung metastases which can be monitored by in vivo imaging (BLI) technology. This GEM model can be an useful source for immunotherapy trials being a model of enhancement of M2-TAMs polarized cells within the TME in primary tumour and lung metastases.

These results are of impact for immunotherapy for studies with new check-points inhibitors with activities against these specialized cells.

**Acknowledgements:** This study was supported by Associazione per la Ricerca sul Cancro IG: 11963 (AIRC-MZ), PRIN (E5AZ5F) 2008 (M.Z.), FP7-Tumic HEALTH-F2-2008- 201662 (M.Z.), Fondazione Adolfo Volpe e Associazione Pediatri di famiglia (M.Z.), POR Rete delle Biotecnologie in Campania Movie (M.Z.), Regione Campania legge n.5 (M.Z.), Wellcome Trust (WT098051) and European School of Molecular Medicine SEMM for the fellowship (V.F.).

**References**

1. Carotenuto M, De Antonellis P, Liguori L, Benvenuto G, Magliulo D, Alonzi A, Turino C, Attanasio C, Damiani V, Bello AM, Vitiello F, Pasquinelli R, Terracciano L, Federico A, Fusco A, Freeman J, Dale TC, Decraene C, Chiappetta G, Piantedosi F, Calabrese C, Zollo M. H-Prune through GSK-3β interaction sustains canonical WNT/β-catenin signaling enhancing cancer progression in NSCLC. Oncotarget. 2014;5(14):5736–49.
2. D’Angelo A, Garzia L, André A, Carotenuto P, Aglio V, Guardiola O, Arrigoni G, Cossu A, Palmieri G, Aravind L, Zollo M. Prune cAMP phosphodiesterase binds nm23-H1 and promotes cancer metastasis. Cancer Cell. 2004;5(2):137–49.
3. Ferrucci V, De Antonellis P, Pennino FP, Asadzadeh F, Virgilio A, Montanaro D, Galeone A, Boffa I, Pisano I, Scogliamiglio I, Diana D, Pedone E, Gargiulo S, Gramanzini M, Brunetti A, Danielson L, Carotenuto MN, Liguori L, Verrico A, Quaglietta L, Errico ME, Del Monaco V, D’Argenio V, Tirone F, Mastronuzzi A, Donofrio V, Giangaspero F, Remke M, Picard D, Garzia L, Daniels C, Delattre O, Swartling JF, Weiss AW, Salvatore F, Fattorusso R, Chesler L, Taylor DM, Cinalli G, Zollo M. Metastatic recurrence of Group 3 medulloblastoma is driven by PRUNE-1 through targeting NM23-H1–TGF-β–OTX2–SNAIL signaling via PTEN inhibition. 2017 **(In press)**.
4. Hashimoto M, Kobayashi T, Tashiro H, Arihiro K, Kikuchi A, Ohdan H. H-Prune is associated with poor prognosis and epithelial–mesenchymal transition in patients with colorectal liver metastases. Int J Cancer. 2016;139(4):812–23.
5. Herschkowitz JI, Simin K, Weigman VJ, Mikaelian I, Usary J, Hu Z, Rasmussen KE, Jones LP, Assefnia S, Chandrasekharan S, Backlund MG, Yin Y, Khramtsov AI, Bastein R, Quackenbush J, Glazer RI, Brown PH, Green JE, Kopelovich L, Furth PA, Palazzo JP, Olopade OI, Bernard PS, Churchill GA, Van Dyke T, Perou CM. Identification of conserved gene expression features between murine mammary carcinoma models and human breast tumors. Genome Biol. 2007;8(5):R76.
6. Hoshino A, Costa-Silva B, Shen TL, Rodrigues G, Hashimoto A, Tesic Mark M, Molina H, Kohsaka S, Di Giannatale A, Ceder S, Singh S, Williams C, Soplop N, Uryu K, Pharmer L, King T, Bojmar L, Davies AE, Ararso Y, Zhang T, Zhang H, Hernandez J, Weiss JM, Dumont-Cole VD, Kramer K, Wexler LH, Narendran A, Schwartz GK, Healey JH, Sandstrom P, Labori KJ, Kure EH, Grandgenett PM, Hollingsworth MA, de Sousa M, Kaur S, Jain M, Mallya K, Batra SK, Jarnagin WR, Brady MS, Fodstad O, Muller V, Pantel K, Minn AJ, Bissell MJ, Garcia BA, Kang Y, Rajasekhar VK, Ghajar CM, Matei I, Peinado H, Bromberg J, Lyden D. Tumour exosome integrins determine organotropic metastasis. Nature. 2015;527(7578):329–35.
7. Li Y, Hively WP, Varmus HE. Use of MMTV-Wnt-1 transgenic mice for studying the genetic basis of breast cancer. Oncogene. 2000;19(8):1002–9.
8. Tammenkoski M, Koivula K, Cusanelli E, Zollo M, Steegborn C, Baykov AA, Lahti R. Human metastasis regulator protein H-prune is a short-chain exopolyphosphatase. Biochemistry. 2008;47(36):9707–13.
9. Zollo M, Andrè A, Cossu A, Sini MC, D’Angelo A, Marino N, Budroni M, Tanda F, Arrigoni G, Palmieri G. Overexpression of H-prune in breast cancer is correlated with advanced disease status. Clin Cancer Res. 2005;11(1):199–205.

#### O5 Characterization of melanoma patients with brain metastases diagnosed between 2014–2016, in one center

##### Teresa Amaral, Ioanna Tampouri, Ulrike Keim, Thomas Eigentler, Claus Garbe, Andrea Forschner

###### Center for Dermatooncology, Department of Dermatology, Liebermeisterstr. 25, University Hospital Tuebingen, 72076 Tuebingen, Germany

*Journal of Translational Medicine 2018*, **16 (Suppl 1):**O5

**Background:** The treatment of patients suffering from melanoma brain metastasis is challenging. Combination of local and systemic therapies is under evaluation in several ongoing clinical trials. Moreover, treatment sequence needs further optimization and therefore standards for the management of brain metastases in melanoma patients do not exist so far.

**Methods:** After approval of the ethical commission, we conducted a retrospective study including 168 patients diagnosed with melanoma brain metastases between 2014 and 2016 and treated with local and/or systemic therapies. The cut-off date for data collection was April 2017. Overall survival was analyzed using a Kaplan–Meier estimator.

**Results:** The median follow-up since the first melanoma diagnosis was 61.8 months [23–80.75] and 8.59 months [3–12] since the first diagnosis of brain metastasis. As of the date cut-off 39% of the patients were still alive. The median melanoma specific survival, defined as the time between melanoma diagnosis and last observation or death, was 63 months [25–129]. The median overall survival (OS) for the all population, defined as the time between brain metastasis diagnosis and last observation or death, was 9 months [4.0–22.0].

For patients treated with immunotherapy as first systemic therapy, the median OS was 13 months (95% CI 7.65–18.35 months) and 11 months (95% CI 6.55–15.46 months; p = 0.005) for those treated with targeted therapy. When the type of first line local therapy is analyzed, the median OS was 22 months (95% CI 11.24–31.76 months) for patients treated with surgery or stereotaxic radiotherapy and 6 months (95% CI 4.36–7.64 months; p = 0.0001) for patients treated with whole brain radiation. The best results were obtained when both systemic and local therapies were combined in a 4 weeks interval, but this was not significant (p = 0.061). In patients with BRAF mutation, longer median OS was observed in patients treated with immunotherapy as first systemic therapy when compared to targeted therapy. The median OS was not reached in the first group and was 11 months (95% CI 6.54–15.45 months; p = 0.004) in the second group.

**Conclusion:** The availability of new therapies increased OS of patients with brain metastases, in comparison with historical controls (9 months vs 5 months). Immunotherapy as first systemic therapy was associated with the best outcomes, including in patients harboring BRAF mutation.

#### O6 A novel translational research tool for the development of predictive signatures of immunotherapy response

##### Alessandra Cesano, Sarah Warren

###### NanoString Technologies, Inc., Seattle, WA, USA

*Journal of Translational Medicine 2018*, **16 (Suppl 1):**O6

The Tumor Inflammation Signature (TIS) is an 18 gene biomarker of a suppressed adaptive immune response within tumor which measures four key areas of biology—antigen presentation, T/NK cell abundance, IFN signaling, and T cell exhaustion. The TIS is currently under evaluation in three clinical trials to predict immune response to pembrolizumab, and may have broad utility to predict response to other immune checkpoint inhibitors. The TIS has been embedded into the NanoString^®^ IO 360 panel—a 770 gene expression panel allows for the parallel assessment of additional mechanisms of immune-evasion in the RUO setting using a single 5 μm FFPE tissue section. The panel contains content to characterize evasion in the context of an inflamed tumor phenotype (such as additional checkpoints inhibitors or suppressive immune cell populations) as well as in the context of an “immune excluded” or “immune desert” tumor microenvironment phenotype (such as activation of oncogenic pathway affecting immune cell trafficking or intrinsic alteration of the antigen presentation process). The IO 360 panel enables the development of diagnostic tests that will select populations that respond to novel and existing immunotherapies as well as combination therapies based on the parallel assessment and integration of multiple mechanisms of immune evasion in a single assay.

### SITC session—evolving topics in cancer immunotherapy

#### O7 Mechanisms of primary resistance to cancer immunotherapies

##### Duane Moogk^1^, Kaitao Li^2^, Zhou Yuan^3^, Shi Zhong^1^, Zhiya Yu^4^, Ivan Liadi^5^, William Rittase^3^, Victoria Fang^1^, Janna Dougherty^1^, Arianne Perez-Garcia^1^, Iman Osman^1,7^, Navin Varadarajan^5^, Nicholas P. Restifo^4^, Alan Frey^6^, Cheng Zhu^2,3^, Michelle Krogsgaard^1,8^

###### ^1^Perlmutter Cancer Center, New York University School of Medicine, New York, NY, USA; ^2^Coulter Department of Biomedical Engineering, Georgia Institute of Technology, Atlanta, GA, USA; ^3^George W. Woodruff School of Mechanical Engineering, Georgia Institute of Technology, Atlanta, GA, USA; ^4^Center for Cancer Research, NCI, NIH, Bethesda, MD, USA; ^5^Department of Chemical and Biomolecular Engineering, University of Houston, Houston, TX, USA; ^6^Perlmutter Cancer Center, Departments of Cell Biology, New York University, New York, NY, USA; ^7^Perlmutter Cancer Center, Departments of Dermatology, New York University, New York, NY, USA; ^8^Perlmutter Cancer Center, Departments of Pathology, New York University, New York, NY, USA

*Journal of Translational Medicine 2018*, **16 (Suppl 1):**O7

**Background:** Although much clinical progress has been made in harnessing the immune system to recognize and target cancer, there is still a significant lack of an understanding of how tumors evade immune recognition and the mechanisms that drive tumor resistance to both T cell and checkpoint blockade immunotherapy. Our objective is to understand how tumor-mediated signaling through inhibitory receptors, including PD-1, combine to affect the process of T cell recognition of tumor antigen and activation signaling, with the goal of understanding the basis of resistance to PD-1 blockade and the potential identification of new molecular targets to enable T cells to overcome dysfunction mediated by multiple inhibitory receptors.

**Methods and results:** We show that Lck activity affects T cell sensitivity and influences the probability of inducing effector function [1]. Under non-activating conditions, Lck and Shp-1 phosphorylation and activity vary based on CD8+ memory T cell phenotype. Shp-1 interaction with Lck under non-activation conditions can also vary, as suggested by our results showing decreased Shp-1 S591 phosphorylation, which affects Shp-1 localization and correlates with increased Shp-1 colocalization with Lck. Further, we showed that Shp-1 directly influences Lck activity under non-activating conditions, as inhibition of Shp-1 leads to increased Lck activity. Importantly, inhibition of Shp-1/2, a major mediator of PD-1 signaling, targeting CD28 and Lck [2], prior to activation leads to increased T cell cytotoxic effector function. Our proteomics-based analysis of patient T cells identified both mediators of PD-1 signaling and signaling components and pathways associated with blockade resistance. It has generally been thought that TCR and CD8 binding depend mainly on their ectodomain interactions with pMHC. We have shown, however, that Lck-CD8 binding [3] and Lck activity [4] are required for upregulated CD8 binding to pre-bound TCR-pMHC complex. Therefore, the cytoplasmic associations of Lck with CD8 and Zap-70, as well as CD3 with Zap-70 may influence formation and stability of the TCR–pMHC–CD8 complex. To determine the mechanistic basis of PD-1 inhibition of TCR–-pMHC–CD8 binding we utilized 2D affinity combined with Biomembrane Force Probe *(BFP)* measurements[5, 6] and showed that PD-1 directly suppresses TCR–pMHC–CD8 binding. Our data also revealed that TCR-pMHC binding was independent of PD-1-PD-L1, but TCR–pMHC–CD8 binding was suppressed by PD-1-PD-L1 binding demonstrating negative cooperativity, as fewer bonds formed than the sum of bonds formed by each interaction alone.

**Conclusions:** Together, our results show that the activities of TCR-proximal signaling components affect T cell mechanosensing and sensitivity at the earliest stages of antigen recognition and are influenced by PD-1. Targeting these interactions may enhance tumor-specific T cell sensitivity for cancer immunotherapy and understanding the basis of resistance to PD-1 blockade to potentially allow identification of new molecular targets to enable T cells to overcome dysfunction mediated by multiple inhibitory receptors.

**References**

1. Moogk D, et al. Constitutive Lck activity drives sensitivity differences between CD8+ memory T cell subsets. J Immunol. 2016 **(In press)**.
2. Hui E, et al. T cell costimulatory receptor CD28 is a primary target for PD-1-mediated inhibition. Science. 2017;355(6332):1428–1433.
3. Casas J, et al. Ligand-engaged TCR is triggered by Lck not associated with CD8 co-receptor. Nat Commun. 2014;5:5624.
4. Jiang N, et al. Two-stage cooperative T cell receptor-peptide major histocompatibility complex-CD8 trimolecular interactions amplify antigen discrimination. Immunity. 2011;34(1):13–23.
5. Evans E, Ritchie K, Merkel R. Sensitive force technique to probe molecular adhesion and structural linkages at biological interfaces. Biophys J. 1995;68(6):2580–7.
6. Huang J, et al. The kinetics of two-dimensional TCR and pMHC interactions determine T-cell responsiveness. Nature. 2010;464(7290):932–6.

#### O8 Role of TGFβ superfamily members in hindering the pro-immunogenic effects of radiotherapy

##### Claire Vanpouille-Box^1^, Silvia C. Formenti^1^ and Sandra Demaria^1^

###### ^1^Department of Radiation Oncology, Weill Cornell Medicine, New York, NY, USA

*Journal of Translational Medicine 2018*, **16 (Suppl 1):**O8

**Background:** Transforming Growth Factor-beta (TGFβ) and activin A (actA) are TGFβ superfamily members with overlapping functions in many processes including regulation of inflammation and immunity. We have recently shown that in situ vaccination by local tumor irradiation is hindered by activation of latent TGFβ [1]. Intriguingly, TGFβ blockade enhanced activation of dendritic cells and T-cell priming, but it increased (rather than reduced) intratumoral regulatory T cells (Tregs). We have recently found that actA release by breast cancer cells is enhanced by radiotherapy (RT). Interestingly, prolonged exposure to TGFβ inhibitors also resulted in actA upregulation, consistent with a previously described compensatory mechanism. Here we hypothesized that actA and TGFβ regulate RT-induced anti-tumor immunity.

**Methods:** Secretion of actA by untreated and irradiated 4T1 mouse carcinoma cells was quantified by ELISA. Transwell co-culture was used to assess the ability of cancer cell-derived actA to promote the conversion of naïve CD4^+^ T cells into Tregs. 4T1 cell derivatives engineered to express a tetracycline-inducible shRNA specific for actA (4T1^shActA^) or non-silencing (4T1^shNS^) were generated and injected s.c. to syngeneic BALB/c mice (day 0). *ActA* knockdown was induced by systemic doxycycline administration at day 8. TGFβ-neutralizing 1D11 or isotype control antibodies were given i.p. every other day starting on day 12. RT was delivered to the primary tumor in 6 Gy fractions on 5 consecutive days beginning on day 13. Mice were followed for tumor growth or euthanized at day 22 for analysis.

**Results:** TGFβ blockade improved RT-mediated tumor control, an effect mediated by T cells. However, tumor recurred. Notably, ActA KD or 1D11 increased intratumoral Tregs (Control: 11%; 1D11: 26%, shActA: 21%) and enhanced Tregs infiltration induced by RT (RT: 15%; RT+1D11: 27%; RT+shActA: 30%). When both TGFβ and actA were blocked Tregs significantly decreased in both untreated (1D11+shActA: 13%) and RT-treated tumors (RT+1D11+shActA: 8% of Tregs). Tumor-specific IFNγ production by CD8+ T cells was significantly higher in RT+1D11+shActA-treated mice compared to RT+1D11 (*p) and RT+shActA (**p). ActA KD in mice treated with RT+1D11 reduced tumor recurrence and improved survival (RT+1D11 vs RT+1D11+shActA **p; RT+shActA vs RT+1D11+shActA ***p).

**Conclusion:** Data indicate that both TGFβ and actA impair RT-induced anti-tumor immune responses. Concomitant inhibition of actA and TGFβ is required for optimal in situ vaccination by RT.

**Reference**

1. Vanpouille-Box C, et al. TGFβ is a master regulator of radiation therapy-induced anti-tumor immunity. Cancer Res 2015;75(11):2232–42.

#### O9 The renin angiotensin system (RAS) mediates bifunctional growth regulation in melanoma and is a target for therapeutic manipulation

##### Cristiana Lo Nigro^1^, Alexander Renziehausen^2^, Andreas G. Tzakos^3^, Hexiao Wang^4^, Bhavya Rao^5^, Rubeta Matin^6^, Catherine Harwood^6^, Daniela Vivenza^1^, Federica Tonissi^1^, Marcella Occelli^1^, Lynda Weir^4^, Su Li^7^, Van Ren Sim^8^, Kevin O’Neill^9^, Alan Evans^10^, Alastair Thompson^11^, Peter Szlosarek^12^, Colin Fleming^13^, Charlotte Proby^6^, Nelofer Syed^1^, Marco Merlano^1^, Tim Crook^14^

###### ^1^Department of Oncology, S. Croce & Carle Teaching Hospital, Cuneo, Italy; ^2^John Fulcher Neuro-Oncology Laboratory, Division of Brain Sciences, Imperial College London, London, UK; ^3^Department of Chemistry, University of Ioannina, Ioannina, Greece; ^4^Department of Dermatology, The First Hospital of China Medical University, Shenyang, China; ^5^Medical Research Institute, Ninewells Hospital & Medical School, Dundee, UK; ^6^Barts and the London School of Medicine and Dentistry, London, UK; ^7^Royal Marsden Hospital, Fulham Road, London, UK; ^8^Kent Oncology Centre, Maidstone Hospital, Maidstone, UK; ^9^Department of Neurosurgery, Charing Cross Hospital, London, UK; ^10^Department of Pathology, Ninewells Hospital, Dundee, UK; ^11^Breast Surgical Oncology, MD Anderson Cancer Center, Houston, Texas, USA; ^12^Department of Medical Oncology, Bart’s Cancer Centre, London, UK; ^13^Department of Dermatology, Ninewells Hospital, Dundee, UK; ^14^Department of Oncology, Royal Surrey County Hospital, Guildford, UK

*Journal of Translational Medicine 2018*, **16 (Suppl 1):**O9

**Background:** Despite the emergence of active new systemic therapies, metastatic melanoma remains a clinically challenging form of skin cancer. The renin-angiotensin system (RAS) is a major physiological regulatory pathway mediated by angiotensin II (AngII) via two receptor subtypes, AT_1_R (encoded by *AGTR1*) and AT_2_R (by *AGTR2*) (1) The role of the RAS is unexplored in melanoma.

**Materials and methods:** We investigated the involvement of the two principal angiotensin receptors in a panel of melanoma cell lines, grown as described previously (2). Primary cultures of brain metastatic melanomas were established from fresh tumour surgical tissues. The selective AT_1_R inhibitor Losartan and the highly selective AT_2_R agonist Y6AII were developed as described (3). Demethylation experiments using azacytidine and trichostatin were done as described (4). TaqMan^®^ probes were used for gene expression analysis. Transfectants with *AGTR1* ORF were analysed for knockdown of *AGTR1* by qPCR and WB. Cell proliferation and clonogenic assays were assessed by standard twchniques. The role of AT_2_R in tumour angiogenesis was investigated in hCMEC/D3 grown in CM collected from PMWK cells treated with AngII alone or in combination with Losartan and PD123319.

**Results:** Antagonism of AT_1_R using the Losartan or *sh*RNA-mediated knock-down in melanoma cell lines expressing *AGTR1* resulted in acquisition of the ability to proliferate in serum-free conditions, implying that AT_1_R has a negative growth-regulatory function in melanoma. Consistent with this, ectopic expression of *AGTR1* in cell lines lacking endogenous expression inhibits proliferation irrespective of the presence of AngII implying a ligand-independent suppressor function for AT_1_R. Treatment of melanoma cell lines expressing endogenous AT_2_R with either AngII or the AT_2_R-specific agonist Y6AII induces proliferation in serum-free conditions. Conversely, the AT_2_R-specific antagonists PD123319 and EMA401 inhibit melanoma growth and angiogenesis and potentiate inhibitors of BRAF and MEK.

Consistent with a negative growth regulatory function, we showed that: (i) decreasing expression and increasing CpG island methylation of *AGTR1* in metastatic vs primary melanoma; (ii) detection in serum of *AGTR1* methylated genomic DNA is associated with metastatic disease.

**Conclusions:** Our results demonstrate that the RAS has both oncogenic and tumour suppressor functions in melanoma. Pharmacological inhibition of AT_2_R may have therapeutic effects in melanomas expressing this receptor and *AGTR1* methylation in serum may serve as a biomarker of metastatic melanoma.

**References**

1. Karnik SS, Unal H, Kemp JR, Tirupula KC, Eguchi S, Vanderheyden PM, et al. Angiotensin receptors: interpreters of pathophysiological angiotensinergic stimuli. Pharmacol Rev. 2015;67:754–819.
2. Hoshimoto S, Kuo CT, Chong KK, Takeshima TL, Takei Y, Li MW, et al. AIM1 and LINE-1 epigenetic aberrations in tumor and serum relate to melanoma progression and disease outcome. J Invest Dermatol. 2012;132:1689–97.
3. Magnani F, Pappas CG, Crook T, Magafa V, Cordopatis P, Ishiguro S, et al. Electronic sculpting of ligand-GPCR subtype selectivity: the case of angiotensin II. ACS Chem Biol 2014;9:1420–425.
4. Miura S, Karnik SS, Saku K. Constitutively active homo-oligomeric angiotensin II type 2 receptor induces cell signalling independent of receptor conformation and ligand stimulation. Journal Biol Chem 2005;280:18237–18244.

#### O10 Functional genomics to identify germline markers of melanoma immunotherapy efficacy and toxicity

##### Robert Ferguson, Danny Simpson, Carlos Martinez, Matjaz Vogelsang, Esther Kazlow, Melissa Wilson, Anna Pavlick, Jeffrey Weber, Ryan Sullivan, Keith Flaherty, Antoni Ribas, Iman Osman, Tomas Kirchhoff

###### Laura and Issac Perlmutter Cancer Center, New York University School of Medicine, New York City, NY, USA

*Journal of Translational Medicine 2018*, **16 (Suppl 1):**O10

**Background:** Approximately 40% of metastatic cutaneous melanoma (CM) patients do not respond to the current immunotherapy (IT) regimens, pointing to other, yet unknown factors conferring IT resistance. In addition, > 60% of patients from single-line or combined treatment (COMBO) regimens present severe immune related adverse events (irAEs). In this study we have developed a novel genomic approach interrogating expression quantitative trait loci (eQTLs) to explore weather germline genetic variation can serve as novel personalized determinant of immunotherapy response and toxicity.

**Methods:** By interrogating the genome wide expression data and SNP array datasets of healthy twin cohort (MuTHER), we have identified 85 eQTLs most significantly associated with the expression of 265 immune genes. Using the MassARRAY system, the 85 SNPs were genotyped in 138 anti-CTLA-4 treated patients, 87 PD-1 treated patients, and 69 patients from combined (COMBO) treatments, collected from multi-institutional collaborations. To test the association of SNPs with IT response and irAEs, logistic regression analyses were performed for each treatment group adjusting by demographic and clinical covariates.

**Results:** We found significant associations with COMBO IT resistance for and eQTL in *IL10/IL19* (OR = 4.249, p = 0.0167), which we have recently identified for association with melanoma survival and which, interestingly, is an established locus associated with the risk of several autoimmune diseases. Additionally, we also identified eQTLs that are associated with IT sensitivity; *IL1*-*β* with resistance to anti-CTLA-4 and *SPI1* with resistance to anti-PD-1. Interestingly, genomic scan of 85 eQTLs has identified novel loci predictive of severe autoimmunity and site specific irAEs in patients treated with COMBO or single-line anti-CTLA4 IT.

**Conclusions:** In this study, we report that eQTLs from *IL19/IL10* locus, previously shown to predict autoimmunity risk and CM survival, is also a surrogate marker of response to COMBO IT, indicating a strong relationship between interleukin pathways and tumor immunogenicity. Novel loci have been found as predictive markers for autoimmune toxicity, in patients treated with COMBO and anti-CTLA4 IT. This is a first evidence that immunomodulatory pathways modulated by germline genetic variation can impact susceptibility to irAEs as well as IT efficacy. Currently, a large scan is underway using genome-wide genetic screens to further test the functional validity of these findings in a large collaborative setting.

### Focus on health cost assessments and pharmacovigilance in immunotherapy

#### O11 Comparison between new therapies of metastatic and/or unresectable melanoma with B-RAF V600E/K mutations: cost–benefit assessment

##### Antonio D’Avino, Licia Guida, Augusto Cosacco, Roberta D’Aniello, Piera Maiolino

###### IRCCS Istituto Nazionale Tumori Fondazione “G. Pascale”, Napoli, Italy

*Journal of Translational Medicine 2018*, **16 (Suppl 1):**O11

**Background:** Incidence of melanoma continues to rise, and the mortality associated with unresectable or metastatic melanoma remains high. B-RAF targeted therapy has been established as a treatment standard for patients who have metastatic melanoma with activating BRAF mutations and recently a regimen combining a B-RAF inhibitor with a MEK inhibitor has been associated with a higher response rate and longer duration of response, as compared with anti-BRAF monotherapies. These drugs are subject monitoring by Italian Medicines Agency (AIFA) and submitted to negotiating agreements between AIFA and Pharmaceutical companies, known as Managed Entry Agreements (MEAs). The objective of our study is to evaluate the cost–benefit evaluation of anti-BRAF and anti-MEK agents alone or in combination to calculate the average of pharmacological cycles performed, the number of progressions and the percentage of partial and/or complete remission percentage Treatments That Meet MEAs.

**Methods:** The clinical, therapeutic and diagnostic data of each patient were obtained from the hospital databases and the monitoring register by Italian Medicines Agency, that contains data for monitoring patients who are receiving this therapies. The report of this register include the medicines that are registered and some of the outcomes that were being monitored, for example, the number of treated patients, the patients that have finalized the treatment and the reasons for stopping the treatment. In these monitoring, we are included all patients with advanced melanoma with B-RAF V600E/K mutation treated between 2012 and 2016.

**Result:** 112 patients with non-resectable metastatic melanoma were treated from 2012 to 2016, of which 68 were treated with monotherapy (65 with Vemurafenib and 3 with Dabrafenib), 44 with BRAF and MEK-inhibitor associations (12 Vemu/Cob and 32 Dab/Tram). Patients performed on average 12 cycles of Vemurafenib, 3 cycles of Dabrafenib, 5 cycles of Vemu/Cob and 6 cycles of Dab/Tram. Compared to the MEAs, over half of the therapies, on average over 60%, monotherapy or association, meet the expectations described.

**Conclusion:** Higher PfS therapies are monotherapy with Vemurafenib (4,4) and the Dabr/Tram (3,26) association, which on average patients take the drug for more time following the Vemurafenib monotherapy (12 cycles), exceeding the MEAs expectations.

**References**

1. American Cancer Society. Melanoma Skin cancer (http://www.cancer.org/acs/groups/cid/documents/webcontent/003120-pdf.pdf).
2. World Health Organization. Ultraviolet radiation and the INTERSUN Programme; skin cancer cancer (http://www.who.int/uv/faq/skincancer/en/index1.html).
3. American Cancer Society. Cancer facts and figures 2014 (http://www.cancer.org.acs/groups/content/@search/documents/webcontent/acspc-042154.pdf).
4. AIOM, CCM, AIRTUM. I numeri del Cancro in Italia 2016. Roma: Il Pensiero Scientifico Editore.
5. Johnston K, Levy AR, Lorigan P, et al. Economic impact of healthcare resource utilisation patterns among patients diagnosed with advanced melanoma in the United Kingdom, Italy, and France: results from a retrospective, longitudinal survey (MELODY study). Eur J Cancer 2012; 48: 2175-82. 10.1016/j.ejca.2012.03.003.
6. Maio M, Ascierto P, Testori A, et al. The cost of unresectable stage III or stage IV melanoma in Italy. J ExpClinCancer Res. 2012;31:91. 10.1186/1756-9966-31-91.
7. Il farmacista nella gestione delle sperimentazioni cliniche: analisi delle sperimentazioni farmacologiche condotte Dal 2011 AL 2014 ALL’ INT. Pascale G, D’Avino A, Maiolino P, D’Aniello R, Sarno MR—Bollettino SIFO 01/2016.
8. Clinical and economic evaluation of the introduction of the combinazion trametinib + dabrafenib in the management of advanced melanoma in the Italian market Lorenzo Pradelli, Paolo Ascierto.
9. Robert C, Karaszewska B, Schachter J, et al. Improved overall survival in melanoma with combined dabrafenib and trametinib. N Engl J Med. 2015;372:30–9.
10. Larkin J, Ascierto PA, Dreno B, et al. Combined vemurafenib and Cobimetinib in BRA-mutated melanoma. N Engl J Med. 2014;371:1867–76.
11. Long GV, Stroyakovskiy D. Gogas H, et al. Combined BRAF inhibition along in melanoma. N Engl Med. 2014;371:1877–88.
12. ClinPharmacokinet. 2017. 10.1007/s40262-017-0523-7. Clinical Pharmacokinetics of Vemurafenib.Zhang W^1^, Heinzmann D^2^, Grippo JF^3^ **(Epub ahead of print)**.
13. Pigment cell melanoma res. 2017;30(1):68–71. 10.1111/pcmr.12557. Pharmacokinetics of dabrafenib in a patient with metastatic melanoma undergoing haemodialysis. Park JJ^1^, Boddy AV^2^, Liu X^2^, Harris D^2,3^, Lee V^2,3^, Kefford RF^1,4,5^, Carlino MS^1,2,5^.
14. Lancet Oncol. 2014;15(9):954–65. 10.1016/S1470-2045(14)70301-8. Epub 2014 Jul 15. Combination of vemurafenib and cobimetinib in patients with advanced BRAF(V600) mutated melanoma: a phase 1b study. Ribas A^1^, et all. J Clin Oncol. 2012;30(15 suppl):8510–8510.
15. Italian Medicines Agency (Agenzia Italiana del Farmaco—AIFA). Registro Farmaci Oncologici sottoposti a Monitoraggio—Rapporto Nazionale 2007. Rome: AIFA. 2008. http://antineoplastici.agenziafarmaco.it/Registro_farmaci.pdf. Accessed 21 Jan 2011.
16. Experiences and Impact of European Risk-Sharing Schemes Focusing on Oncology Medicines Jaime Espín, Joan Rovira and Leticia García Andalusian School of Public Health. 2011.
17. Flaherty KT, Infante JR, Daud A, et al. Combined BRAF and MEK inhibition in melanoma with BRAF V600 mutations. N Engl J Med. 2012;367:1694–703.
18. Long GV, Stroyakovskiy D, Gogas H, et al. Combined BRAF and MEK inhibition versus BRAF inhibition alone in melanoma. N Engl J Med. 2014;371:1877–88.
19. Robert C, Karaszewska B, Schachter J, et al. Improved overall survival in melanoma with combined dabrafenib and trametinib. N Engl J Med. 2015;372:30–9.

#### O12 The drug day an effective strategy for the containment of immunotherapy costs

##### Teresa Tramontano, Maria R. Sarno, Ida Palazzo, Angela Di Napoli, Gianclaudio Acunzo, Piera Maiolino

###### S.C. Farmacia, IRCCS Fondazione “G. Pascale”, Napoli, Italy

*Journal of Translational Medicine 2018*, **16 (Suppl 1):**O12

**Background:** The Centralized Unit for Handling Antineoplastic of National Cancer Institute “G. Pascale” of Naples planned a strategy for the use of high cost cancer drug Opdivo^®^. In order to reduce the therapy cost we decide to dedicate for Opdivo^®^ a drug day to optimize the use of drug vials with reduction of waste and/or optimal use of the residues. The aim of this work was to report the analysis of drug consumption and cost from March 2016 when it was officially authorized as a hospital drug and it was introduced into clinical practice to August 2017.

**Materials and methods:** The drug day was organized in accord with Opdivo^®^ prescribed physicians. The number of patients treated, the individual patient’s treatment line, consumption and cost data relating to the period under review, were collected from the hospital database. In primis, it was performed an assessment of the total consumption and cost per year and then a comparison exercise was made between the number of drug vials really used and those that should be used without the drug day and, the comparison between the real cost incurred and the hypothetical cost out of drug day.

**Results:** From March 2016 to August 2017 the pharmacy staff set 1872 preparation for 211 patients of Opdivo^®^. The drug was administered for 68 patients as first-line treatment, for 116 patients as second-line treatment and finally for 27 patients as third-line treatment. Given the considerable number of patients and considering that Opdivo^®^ stability is 24 h, 2 consecutive days per week were dedicated for treatment as drug days with a range of 15–20 preparations per day. Comparing the used drug in drug day to a hypothetical daily preparation we saved 36.860 mg (approximately 367 vials of 100 mg) with an economy of €435.943,22. Noteworthy, the overfill of injecting drug vials, corresponding to about 10–12 mg over the declared amount of drug, as prescribed by F.U. XII Edition (2.9.17) and by FDA guidelines on the filling volume in excess of vials, allowed us to rescue 13.167 mg with an economy of €155.887,67.

**Conclusions:** Programming cancer therapies in a drug day was very complex and involved close cooperation between prescribed physicians, pharmacists and patients. However this strategy allows to reduce at minimum drug waste residues and, furthermore, to use overfill of the samples, which should became waste. In conclusion the drug day resulted a very effective tool for the containment of pharmaceutical costs.

#### O13 Adverse effects related to PD-1 inhibitor immunotherapy: an overview

##### Mariarosanna De Fina

###### Department Science of Life, Magna Graecia University, Catanzaro, Italy

*Journal of Translational Medicine 2018*, **16 (Suppl 1):**O13

**Background:** Immunotherapies, like nivolumab (Opdivo^®^) and pembrolizumab (Keytruda^®^), have changed the cancer treatment landscape. They are inhibitors of PD-1 protein, which cancer cells use to elude the immune system. Post-marketing surveillance of these drugs have revealed severe adverse drug reactions (ADRs). The occurrence of ADRs has high morbidity and mortality, accounting for the fifth leading cause of death in industrialised countries. EudraVigilance.org is European Medicine Agency’s (EMA) web-based Monitor System for reporting and evaluating suspected ADRs.

**Aim:** A retrospective observational study was done. The aim of this study is to investigate the ADRs occurred in patients treated with PD-1 inhibitors.

**Methods:** All ADRs associated with Opdivo^®^ and Keytruda^®^ reported in EudraVigilance up to 24 September 2017 were analysed for overall numbers, age, gender and geographic origin. A quantitative measurement (proportional reporting ratio [PRRs]) was developed for signal generation from large databases of spontaneous ADRs reports to find a statistical link between each PD-1 inhibitor and ADRs.

**Results:** A total of 10600 ADRs related to the PD-1 Inhibitors therapy have been reported and analysed in this study. Opdivo (65.87%), Keytruda (31.5%) and nivolumab (2.63%). 55.25% occurred in males (male/female ratio = 1.73). Only in 12.8% of ADRs reported, sex is not specified. Most ADRs occurred in patients with age between 18 and 65 years (41.6%). The higher incidence of ADRs occurred in patients of non-European Economic Area than European Economic Area, respectively 73.7% vs 26.3%.

The “Neoplasm benign malignant and unspecified” is the most reported ADRs category for both Opdivo^®^ and Keytruda^®^ (18.9% vs 27.7%) with a PRR = 0.67, ADRs occurred in males show PRR = 8.76. Serious but rare ADRs reported in database is Uveitis, that shows PRR = 0.61. The Uveitis incidence rate, reported in database, is 0.70 vs 0.33, respectively for Keytruda^®^-treated patients than Opdivo^®^-treated patients.

**Conclusions:** Analysis of Pharmacovigilance database is important to understand the safety of drugs in post-marketing and in real clinical practice. Patient safety is priority in care management. For this reason, oncologist, pharmacists and multidisciplinary team have to be careful about adverse reactions that could be occurred in patients treated.

Limitations of this study was cases were based on spontaneous reporting which clearly suffered from underreporting. Clinical data were not available.

## Immunotherapy bridge 2017—poster presentations

### P1 Excellent response to anti-PD-1 therapy in a patient with metastatic malignant melanoma: case report

#### Antonia Silvestri, Monica Specchia, Michela Musacchio, Gianfranco Giglio, Francesco Carrozza, Liberato Di Lullo

##### U.O.C. Oncologia Medica, Ospedale “A. Cardarelli”, Campobasso, Italy

*Journal of Translational Medicine 2018*, **16 (Suppl 1):**P1

**Background:** Malignant melanoma is an aggressive cancer associated with high mortality worldwide. Many patients have locally advanced or metastatic disease at diagnosis in which case treatments were limited until a few years ago. Immune checkpoint blockade of programmed death receptor-1 (PD-1) pathway represents a recent valid treatment strategy changing the prognosis in this setting.

**Case presentation:** Here we describe the case of a 83-year-old man with many comorbidities (chronic renal failure, diabetes, hypertension) and with colorectal cancer anamnesis (right hemicolectomy in 2004) and laryngeal neoplasia (total laryngectomy in 1981) who presented initially neoformation of the parotid region and a skin nodule of the temporal region at right side.

**Results:** Histological examination on both samples showed epithelial malignant melanoma, BRAF wild type. At CT imaging there was the parotid neoformation extended in the regional adipose tissue predominantly, into contact with the right masseter muscle and with the lower wall of the outer external conduit cranially.

In both lungs there were some parenchymal lesions compatible with secondary localizations. On 8 April 2016 the patient started NIVOLUMAB 300 mg total/2 weeks. After the first administration there was a reduction in skin lesions and after 2 months skin nodule of the temporal region were disappeared as well as neoformation of the parotid region. On October at CT imaging, the alterations to the parotid region were completely disappeared and lung metastases appeared hyperdense and cavitated. Last total body PET/TAC (on February 2017) described a single pulmonary localization to the left basal pyramid. The patient is continuing nivolumab therapy with good tolerability, with the exception of a skin rash G1-2 and he is still being in response at a distance of more than 1 year.

**Conclusions:** This case report confirms the efficacy of nivolumab in metastatic melanoma and the safety in elderly patients with polypathology also.

**Consent for publication:** The authors declare that written informed consent was obtained from the patients for publication.

#### P2 Pharmacovigilance in melanoma immunotherapy

##### Gian Marco De Maddi, Adele Venturelli

###### ^1^Pharmacy S.Giovanni Bosco Hospital A.S.L. Napoli 1 Centro, Napoli, Italy; ^2^Pharmacovigilance Referent A.S.L. Napoli 1 Centro, Napoli, Italy

*Journal of Translational Medicine 2018*, **16 (Suppl 1):**P2

**Background:** Ipilimumab, Nivolumab and Pembrolizumab recently approved by the Italian Medicines Agency for first-line therapy of melanoma. They are active on the immune system and have a toxicity related to their mechanism of action. Skin, gastrointestinal tract, liver, endocrine system are mainly affected. Additional monitoring is planned for Nivolumab and Pembrolizumab to allow rapid acquisition of new data about their safety [1, 2]. The aim of Authors is to assess whether this post-authorization monitoring has so far provided about updating the risk profile of these inhibitors of immune checkpoints.

**Materials and methods:** Suspected adverse reactions (ADRs), associated with the use of Ipilimumab, Nivolumab and Pembrolizumab in the treatment of melanoma and recorded in the Pharmacovigilance National Network in the 2015–2016 biennium, were examined and compared with the side effects reported in the Product Characteristics Summary of these drugs and the literature found in Pubmed-Medline and Cochrane Library.

**Results:** From 1 January 2015 to 31 October 2016 111 ADRs were found: 93 relate Ipilimumab, 6 Nivolumab and 12 Pembrolizumab. They were evaluated 57 reports and others could not be classified; diarrhea was the main adverse event reported and 23 ADRs were serious. Three cases only were treated with systemic immunosuppressive drugs. Analysis of adverse reactions showed that they are common side effects of three inhibitors of immune checkpoints.

**Conclusions:** The pharmacovigilance of Ipilimumab, Nivolumab and Pembrolizumab has produced an appreciable number of reports which however represent known side effects of recent melanoma immunotherapy that can be properly treated with systemic immunosuppressive drugs if recognized early [3]. Severity of adverse events has generally required hospitalization: recognition and treatment of these reactions may have been inappropriate or later. Authors believe not all health workers were joined by a clear and complete information about adverse events of these drugs, their recognition and treatment. In cooperation with the oncologist, hospital chemist is able to promote informational programs that can guarantee the knowledge necessary for an adequate approach to toxicity associated to use of these drugs. The aim is ensuring an effective and efficient health service; in this case a correct information can produce a higher yield by reducing costs associated to inappropriate therapies and excessive hospitalization.

**References**

1. Agenzia Italiana del Farmaco Riclassificazione del medicinale per uso umano « Opdivo » , ai sensi dell’articolo 8, comma 10, della legge 24 dicembre 1993, n. 537. Determina AIFA n. 378 dell’11 marzo 2016. Gazzetta Ufficiale n. 70 del 24-3-2016.
2. Agenzia Italiana del Farmaco Riclassificazione del medicinale per uso umano « Keytruda » , ai sensi dell’articolo 8, comma 10, della legge 24 dicembre 1993, n. 537. Determina AIFA n. 589 del 22 aprile 2016. Gazzetta Ufficiale n. 108 del 10-5-2016.
3. Bourke JM, O’Sullivan M, Khattak MA. Management of adverse events related to new cancer immunotherapy (immune checkpoint inhibitors). Med J Aust. 2016.

#### P3 Bullous pemphigoid during Nivolumab therapy: a case report

##### Elisabetta Gambale, Alessia Gatta, Davide Brocco, Consiglia Carella, Michele De Tursi

###### ^1^Department of Medical, Oral and Biotechnological Sciences, Medical Oncology Unit, “G. D’Annunzio” University, Chieti, Italy; ^2^Department of Medicine and Science of Ageing, “G. D’Annunzio” University, Chieti, Italy

*Journal of Translational Medicine 2018*, **16 (Suppl 1):**P3

**Background:** Anti-programmed cell death protein-1 (PD-1)/ligand-1 (PD-L1) antibodies can induce an immune-related bullous pemphigoid (BP) [1, 2]. About its pathogenesis, it has been hypothesized that the blockade of the PD-1/PD-L1 pathway may increase autoantibody production against the hemidesmosomal protein BP180 [1]. Here we report a case of BP during treatment with nivolumab.

**Fig. 1:**
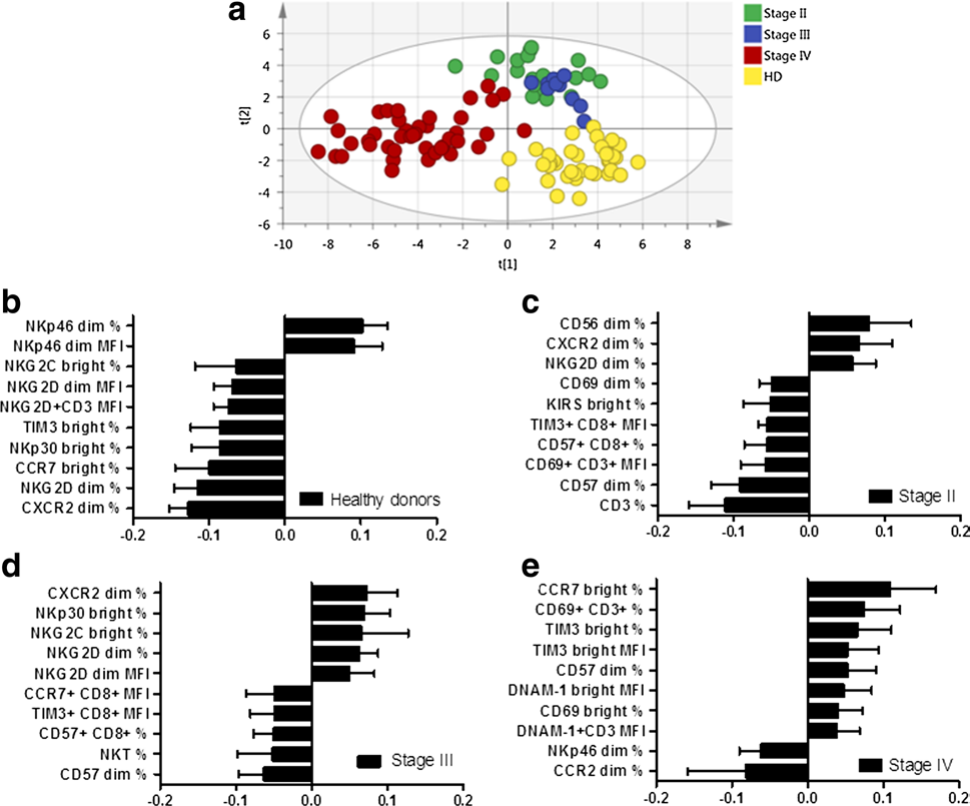
Discriminant analysis of healthy donors and melanoma patients

**Case report:** A 68-year-old man was admitted to our Oncology Unit in March 2015 with the diagnosis of BRAF wild-type melanoma. A CT scan performed on May 2016 showed lung metastases. Patient’s medical history included hypertension. Long-standing medications included omeprazole and zofenopril. He had no relevant history of skin or autoimmune disorders and no new medications. On June 2016, nivolumab was started. On June 2017, the patient began to develop erosions and vesicles on the buccal mucosa, especially with the involvement of the lower lip. The severity of the patient’s bullous dermatitis ranged from grade 1–2. A biopsy of these lesions showed eosinophilic spongiosis and a mixed dermal inflammatory infiltrate with eosinophils. Direct immunofluorescence showed linear deposition of IgG and C3 at the basement of the dermoepidermal junction, establishing a diagnosis of BP. A treatment with oral prednisone dosed according to severity and topic clobetasol was started. He improved within about 2 weeks and the steroid was tapered (Fig. 1). Nivolumab was restarted. Total IgE level was elevated and complete blood count revealed an increased absolute eosinophil count compared to pre-treatment levels, but it remained within normal ranges. The patient experienced a near-complete response to nivolumab, how shown by CT scan performed on August 2017. At this time, he presents only some lesions in the lower lip and he continues treatment with prednisone 5 mg bid and intermittent topical steroids.

**Conclusion:** Although we cannot be sure that BP is due to nivolumab, the timing of cancer diagnosis (24 months prior) compared with nivolumab initiation (12 months prior) and the fact that the other medications were tolerated for years without similar cutaneous lesions argue against a paraneoplastic BP or BP related to another medication, respectively [1,2]. About treatment of BP during anti-PD-1/PD-L1, our research in the literature found a case report of BP during nivolumab with elevated IgE level and successfully treated with omalizumab, thus sparing steroids that could interfere with the antitumor activity of nivolumab [2,3]. Finally, all but few BP cases in the setting of anti-PD-1/PD-L1 therapy were associated with partial/complete response or stable disease: is there a relationship between development of BP and anti-cancer responses [1,2]?

**Consent for publication:** The authors declare that written informed consent was obtained from the patients for publication.

## Melanoma bridge 2017—Keynote speaker presentations

### System biology session in melanoma

#### K4 Tumor and host genomics regulating immunotherapy efficacy

##### Thomas F. Gajewski

###### Department of Pathology and Department of Medicine (Section of Hematology/Oncology)-University of Chicago, Microbiome Center, Chicago, Illinois, US

*Journal of Translational Medicine 2018*, **16 (Suppl 1):**K4

Two major phenotypes of human melanoma metastases have been observed based on gene expression profiling and confirmatory assays. One subgroup of patients has a T cell-inflamed phenotype that includes expression of chemokines, T cell markers, and a type I IFN signature. In contrast, the other major subset lacks this phenotype and appears to display immune “exclusion”. The mechanisms of immune escape are likely distinct in these two subsets, and therefore the optimal immunotherapeutic interventions necessary to promote clinical responses may be different. The T cell-inflamed tumor microenvironment subset shows the highest expression of negative regulatory factors, including PD-L1, IDO, and FoxP3+ Tregs. Deep analysis of tumor antigen-specific T cells in the tumor microenvironment has identified additional mechanisms of immune dysfunction and new potential therapeutic targets. Treatment strategies targeting several pathways have been translated back into the clinic, including anti-PD-1/PD-L1 mAbs and IDO inhibitors, and combinations of these agents also look promising. In contrast to the T cell-inflamed melanomas, non-T cell-inflamed tumors are largely immunotherapy resistant with current approaches. Natural innate immune sensing of tumors appears to occur via the host STING pathway, type I IFN production, and cross-priming of T cells via CD8+ DCs, and these factors are absent in non-T cell-inflamed tumors. New strategies are being developed to engage or mimic this pathway as a therapeutic endeavor, including STING agonists. The molecular mechanisms that mediate the absence of the T cell-inflamed tumor microenvironment in patients are being elucidated using parallel genomics platforms. The first oncogene pathway identified that mediates immune exclusion is the Wnt/β-catenin pathway, which argues that new pharmacologic strategies to target this pathway should be developed to restore immune access to the tumor microenvironment. Recent evidence has indicated that host factors, including the intestinal microbiota, are also critical. We recently have identified commensal bacteria in mouse models that augment spontaneous anti-tumor immunity and increase efficacy of anti-PD-L1 therapy. Similar analyses in human melanoma patients have been performed, and commensal bacteria have similarly been identified that correlate with anti-PD-1 efficacy. These results have prompted the development of new probiotics as potential therapeutics, to improve spontaneous immune infiltration and expand immunotherapy efficacy in the clinic.

## Melanoma bridge 2017—oral presentations

### System biology session in melanoma

#### O14 Monitoring of melanoma clinical progression by circulating NK and T cells immunoprofiling: a potential role for CCR7 in metastatic spread

##### Costanza M. Cristiani^1^, Rossana Tallerico^1^, Valeria Ventura^1,5^, Mariaelena Capone^3^, Gabriele Madonna^3^, Domenico Mallardo^3^, Eliska Selinger ^1^, Cinzia Garofalo^1^, Elina Staaf^2^, Ester Simeone^3^, Antonio M. Grimaldi^3^, Genny del Zotto^4^, Elio Gulletta^5^, Gennaro Ciliberto^6^, Alessandro Moretta^7^, Paolo A. Ascierto^3^ and Ennio Carbone^1,2^

###### ^1^Tumor Immunology and Immunopathology Laboratory, Department of Experimental and Clinical Medicine, University “Magna Græcia” of Catanzaro, Campus-Germaneto, Catanzaro, Italy; ^2^Department of Microbiology, Cell and Tumorbiology (MTC), Karolinska Institutet, Stockholm, Sweden; ^3^Melanoma Cancer Immunotherapy and Innovative Therapy Unit, Istituto Nazionale Tumori Fondazione “G. Pascale”, Naples, Italy; ^4^Istituto Giannina Gaslini, Genova, Italy; ^5^Department of Health Sciences, University “Magna Græcia” of Catanzaro, Campus-Germaneto, Catanzaro, Italy; ^6^Scientific Directorate, IRCCS Istituto Nazionale Tumori Fondazione “G. Pascale”, Naples, Italy; ^7^Department of Experimental Medicine and Center of Excellence for Biomedical Research, University of Genoa, Genova, Italy

*Journal of Translational Medicine 2018*, **16 (Suppl 1):**O14

**Background:** Natural Killer (NK) cells selectively recognize lymph node associated melanoma metastasis cells [1]. Peripheral blood frequencies of CD56bright and CD56dim NK cell subsets are subverted in stage III melanoma patients, while a high frequency of CCR7+KIR+CD57+CD56dim NK cells in melanoma colonized lymph nodes directly correlates with the patients survival [2]. Here, we validate and identify additional changes in the NK cells repertoire characterizing the transition from the different stages of melanoma that can improve the patients diagnosis, and dissected CCR7 potential role in metastatic process by measuring it on both melanoma and NK cells.

**Fig. 2:**
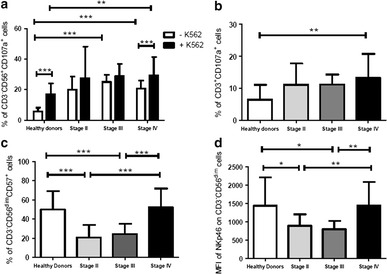
NK and T cells function analysis in healthy donors and melanoma patients

**Results:** Immuno-profile of 42 healthy donors and 103 melanoma patients (stage II, III, IV), together with biographical variables, was used to create an OPLS-DA multivariate model. The model gave a good separation between healthy donors and the three groups of patients (Fig. 1A). Immuno profile of both stage II and III melanoma patients showed an increased CXCR2 percentage, as previously observed [2], and a reduced CD57 frequency and NKp46 expression on the NKdim cells, that correlated with lack of responsiveness to K562 cells pulsing (Figure 2). Instead, stage IV melanoma patients showed high frequencies of, CCR7+CD56bright NK cells, which displayed a longitudinal steady increase during the disease evolution.

Cytokine profile analysis showed a progressive sera accumulation of MCP-1, IL-6, IL-8 IL-15 and CCL19 (Table 1), with the first three that have been demonstrated to be produced by primary melanoma cells and found in TILN milieu [2], while IL-15 was involved in phenotypic changes of NK cells from melanoma patients [3]. CCL19 longitudinal increase over the clinical evolution, perfectly matched with the accumulation of circulating CD56brightCCR7+ cells subset (Table 1).

**Table 1:** Univariate analysis of melanoma patients sera cytokines

Cytokine	Physiological range (pg/ml)	Mean ± SD stage II (pg/ml)	Mean ± SD stage III (pg/ml)	Mean ± SD stage IV (pg/ml)	p value
MCP1	70.5–203	232.1 ± 54.0	225.4 ± 69.1	379.7 ± 222.9	0.0085
IL-6	0–5.6	1.9 ± 2.2	1.1 ± 1.0	12.1 ± 14.6	< 0.0001
IL-8	1.9–17.4	9.1 ± 8.1	13.7 ± 18.3	49.6 ± 51.7	0.0013
IL-15	0–3.9	2.0 ± 0.3	1.7 ± 0.5	4.0 ± 1.9	< 0.0001
CCL19	N. T.	164.9 ± 132.5	58.6 ± 73.1	820.8 ± 403.6	0.0490

**Fig. 3:**
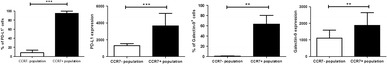
PD-L1 and Tim-3 frequency and expression on CCR7− (white bars) and CCR7+ (Black bars) melanoma cells

CCR7 analysis on melanoma cells showed that it is expressed by a small fraction (1–5%), which is further characterized by a selective co-expression between CCR7 and the inhibitory ligands PD-L1 and Galectin-9 (Fig. 3).

**Conclusions:** Our data suggest a role of CCR7 expression on both melanoma and NK cells that can explain the lymph node tumor colonization and the conversion of the melanoma lesion from local to systemic.

**References**

1. Burke S, Lakshmikanth T, Colucci F, Carbone E. New views on natural killer cell-based immunotherapy for melanoma treatment. Trend Immunol. 2010;31(9):339–45.
2. Ali TH, Pisanti S, Ciaglia E, Mortarini R, Anichini A, Garofalo C, Tallerico R, Santinami M, Gulletta E, Ietto C, Galgani M, Matarese G,7, Bifulco M, Ferrone S, Colucci F, Moretta A, Kärre K, Carbone E. Enrichment of CD56(dim) KIR+ CD57+ highly cytotoxic NK cells in tumour-infiltrated lymph nodes of melanoma patients. Nat. Commun. 2014;4(5):5639.
3. Tallerico R, Cristiani CM, Staaf E, Garofalo C, Sottile R, Capone M, Pico de Coaña Y, Madonna G, Palella E, Wolodarski M, Carannante V, Mallardo D, Simeone E, Grimaldi AM, Johansson S, Frumento P, Gulletta E, Anichini A, Colucci F, Ciliberto G, Kiessling R, Kärre K, Ascierto PA, Carbone E. IL-15, TIM-3 and NK cells subsets predict responsiveness to anti-CTLA-4 treatment in melanoma patients. Oncoimmunology. 2016;6(2):e1261242.

#### O15 Outcome prediction on melanoma patients subjected to immunotherapy treatments by 1H-NMR metabolomic profiling approach

##### Susan Costantini^1^, Angela Sorice^1^, Francesca Capone^1^, Gabriele Madonna^2^, Domenico Mallardo^2^, Mariaelena Capone^2^, Gennaro Ciliberto^3^, Alfredo Budillon^1^, Paolo A Ascierto^2^

###### ^1^Experimental Pharmacology Unit, Istituto Nazionale Tumori Fondazione “G. Pascale”-IRCCS, Napoli, Italy; ^2^Melanoma Cancer Immunotherapy and Innovative Therapy Unit, Istituto Nazionale Tumori “Fondazione G. Pascale” IRCCS, Napoli, Italy; ^3^Scientific Directorate, Istituto Nazionale Tumori “Regina Elena”, IRCCS, Roma, Italy

*Journal of Translational Medicine 2018*, **16 (Suppl 1):**O15

**Background:** The evaluation of metabolomic profiling in biological fluids is recently emerged as a powerful and reliable tool for the identification of novel biomarkers to improve early diagnosis and prognosis classification, as well as prediction of treatment benefit in cancer patients. In this context, Nuclear Magnetic Resonance (NMR) spectroscopy represents the only non-destructive technique able to identify and quantify complex mixtures of metabolites by using small sample volumes and a fast sample preparation approach. In our laboratory we performed metabolomic profiling on liquid biopsy samples collected in eighty metastatic melanoma patients subjected to different immunotherapy treatments (i.e. anti-PD1 MoAb nivolumab and pembrolizumab, anti CTL4 MoAb ipilimumab).

**Materials and methods:** A 600 MHz Avance Bruker spectrometer with cryoprobe, equipped with an automation system was used to acquire 1H-NMR spectra on all samples at 300 K. The spectral 0.50–8.60 ppm region of the 1H-NMR spectra was integrated in buckets of 0.04 ppm using the AMIX package. The bucketed regions were normalized to the total spectrum area using Pareto scaling. Orthogonal Projections to Latent Structures Discriminant Analysis (OPLS-DA) and Loading Plot were used by the MetaboAnalyst tool to analyze and to compare the different groups.

**Results:** The patients were subdivided in two sub-groups to obtain a training set and a validation set. Hence, firstly 1H-NMR spectra were acquired and analyzed on samples belonging to training set. Through this procedure, we were able to group in separate clusters the patients with different outcome in terms of overall survival and to identify a set of metabolites that can discriminate patients before treatments with favorable than those with worst outcome. Then, we validated the significance of the selected metabolites in the validation set. Finally, ROC plots and Kaplan–Meier curves evidenced the metabolites correlated with overall survival.

**Conclusion:** Overall, these data suggest that metabolomic profiling performed on liquid biopsy samples by NMR is a potent and affordable method to improve outcome prediction for melanoma treatment.

#### O16 MiRNA analysis as a complement to screening procedures for early diagnosis of Malignant Melanoma (MM)

##### Marcella Occelli^1^, Carolina Cauchi^1^, Grazia Sciancalepore^2,^, Cristiana Lo Nigro^1^, Michela Rovera^3^, Chiara Varamo^7^, Daniela Vivenza^1^, Federica Tonissi^1^, Zelda Seia^4^, Stefania Palazzini^4^, Fabiana Errico^4^, Davide Basso^4^, Laura Quaranta^4^, Giuseppe Forte^2^, Fulvio Lavagna^5^, Silvia Violante^1^, Paolo Bosio^6^, Laura Lattanzio^1^, Marco C Merlano^1^

###### ^1^Oncologia medica-AO Santa Croce E Carle, Cuneo, Italy; ^2^anatomia Patologica-AO Santa Croce E Carle, Cuneo, Italy; ^3^CAS–AO Santa Croce E Carle, Cuneo, Italy; ^4^LILT, Cuneo, Italy; ^5^SS Day Surgery- AO Santa Croce E Carle, Cuneo, Italy; ^6^Chirurgia Generale- AO Santa Croce E Carle, Cuneo, Italy; ^7^Oncologia–IRCCS, Candiolo, Italy

*Journal of Translational Medicine 2018*, **16 (Suppl 1):**O16

**Background:** MM is the most aggressive skin cancer, its incidence doubled over the past 10 years and its mortality is still around 80%, in the advanced disease. The goal of the present study is to find new more effective screening methods, and to identify MM at high risk of recurrence [1, 2]. In more details, the aim of the project is to investigate the role of specific miRNAs, as a screening tool of MM [3- 6].

**Materials and methods:** Individuals who joined the melanoma screening at the “Lega italiana per la lotta contro i tumori” (LILT), and with a suspect skin lesion, after signing informed consent, underwent a peripheral blood sample collection before the surgical removal.

We compared the plasma level of 15 miRNAs in confirmed MM patients, versus non-confirmed. The study planned to enrol 700 subjects, to identify 100 MM.

The considered miRNAs (miR-15b, miR-122, miR-126, miR-150, miR-155, miR-182, miR-199, miR-200c, miR-205, miR-211, miR-23b, miR- 33a, miR-34a, miR-424 and miR-29c) were selected on the basis of literature data and measured by RT-PCR.

**Results:** From September 2014 to December 2016, 16% (101/633) of enrolled individuals had histologic confirmation of MM: 38 in situ and 59 infiltrating, instead 4 patients were ineligible and were excluded from analysis; Among the patients without MM confirmation, we selected 100 controls who best matched the characteristics of the experimental population.

Till now we have analysed the role of miRNAs as potential tools for screening, while their prognostic role is not yet evaluated. The analysis showed a significant reduction in the expression of miR-199a-5p (p = 0.0027) and miR-122-5p (p = 0.05) in the MM (N = 97) group compared to the controls (N = 100). Multivariate analysis is underway in order to associate miRNA expression profiling with clinical-pathological characteristics.

**Conclusions:** The data available today do not allow to identify a miRNA that can be used as a complementary tool for the screening of early-stage MM. Significant reduction of miR-122-5p and miR199-5p in MM compared to controls, however, leads us to deepen their biological role in the pathogenesis of MM and their possible association with clinical-pathological features of subjects under screening. Multivariate analysis data will be available and presented at the meeting.

**Acknowledgements:** This project has been realized with the support of the CRC Foundation and the LILT section of Cuneo.

**References**

1. AIOM Airtum: i numeri del cancro in Italia, 2016, intermedia ed. Brescia.
2. Duff CG, et al. A 6 year prospective analysis of the diagnosis of malignant melanoma in pigmented-lesion clinic: even the experts miss malignant melanoma, but not often. Br J Plast Surg. 2001;54:317–21.
3. Kasinski AL, et al. Epigenetics and genetics. MicroRNAs en route to the clinic: progress in validating and targeting microRNAs for cancer therapy. Nat Rev Cancer. 2011;11:849–864.
4. Kanemaru H, et al. The circulating microRNA-221 level in patients with malignant melanoma as a new tumor marker. J Dermatol Sci. 2011;61(3):187–93. 10.1016/j.jdermsci.2010.12.010. Epub 2011 Jan 13.
5. Leidinger, et al. Hight-throughput miRNA profiling of human melanoma blood samples. BMC Cancer. 2010;10:262.
6. Friedman EB, et al. Serum microRNAs as biomarkers for recurrence in melanoma. J Transl Med 2012;10:155.

#### O17 Predictive biomarkers of check point inhibition toxicity in metastatic melanoma

##### Mike Gowen^1^, Jeremy Tchack^1^, Hua Zhou^2^, Keith Giles^1^, Scott Paschke^3^, Una Moran^1^, David Fenyo^2^, Aris Tsirigos^2^, Michael Pacold^4^, Anna Pavlick^5^, Michelle Krogsgaard^6^, Iman Osman^1,5^

###### ^1^The Ronald O. Perelman Department of Dermatology, New York University School of Medicine, New York, NY, USA; ^2^Center for Health Informatics and Bioinformatics, NYU Langone Medical Center, New York, NY, USA; ^3^CDI Laboratories, Baltimore, MD, USA; ^4^Department of Radiation Oncology, NYU School of Medicine, New York, NY, USA; ^5^Division of Hematology & Oncology, Laura and Isaac Perlmutter Cancer Center, New York, NY, USA; ^6^Department of Pathology, NYU School of Medicine, New York, NY, USA

*Journal of Translational Medicine 2018*, **16 (Suppl 1):**O17

**Background:** There are no predictive biomarkers of ipilimumab (IPI) toxicity. Of metastatic melanoma (MM) patients (pts) receiving IPI (3 mg/kg), 35% require systemic therapies to treat immune-related adverse events (irAEs) and 20% must terminate treatment [1]. Here we tested the hypothesis that a pre-existing autoantibody (autoAb) profile is predictive of IPI irAEs.

**Methods:** We measured autoAb levels in pre- and post-treatment sera from MM pts who received IPI (3 mg/kg) monotherapy on a proteome microarray containing ~ 20,000 unique full-length human proteins (HuProt array, CDI Laboratories). Clinical data were prospectively collected with protocol-driven follow-up. IrAEs were categorized by CTCAE guidelines as none (grade 0), mild (grade 1–2), or severe (grade 3–4). AutoAb levels were standardized using median quantile normalization and considered positive hits if > 2-SD above the peak array signal and differed by ≥ 2-fold with p < 0.05 between toxicity groups (Non-parametric Analysis/Wilcox test).

**Results:** Seventy-eight sera from 37 MM pts were analyzed. Antibodies against CTLA-4 were significantly elevated post IPI treatment (p < 0.0001), validating the assay. The pre-treatment levels of 190 IgG autoAbs were significantly different in pts who experienced irAEs (n = 28) compared to those with no irAEs (n = 9). Comparison of severe irAE (n = 9) and no irAE (n = 9) groups revealed 129 IgG autoAbs that significantly differed in pre-treatment sera. Localization and pathway analysis (UniProt, KEGG, Reactome) showed 81/190 (43%) of the autoAbs targeted nuclear and mitochondrial antigens and were enriched in metabolic pathways (p = 0.015). AutoAbs associated with irAEs did not correlate with treatment response.

**Conclusions:** AutoAbs to antigens enriched in metabolic pathways prior to treatment may predict IPI-induced toxicity in MM. The subcellular localization of targeted antigens could explain the autoimmune toxicities associated with IPI. Studies in larger cohorts and in pts receiving other checkpoint inhibitors and/or combination therapies are essential to determine the validity of the data. If validated, our results would support the discovery of the first toxicity predictor in cancer immunotherapy.

**Reference**

1. Horvat TZ, Adel NG, Dang T-O, et al. Immune-related adverse events, need for systemic immunosuppression, and effects on survival and time to treatment failure in patients with melanoma treated with ipilimumab at memorial sloan kettering cancer center. J Clin Oncol. 2015;33(28):3193–3198.

#### O18 Soluble CD73 in the peripheral blood: a potential biomarker in patients with advanced melanoma receiving nivolumab

##### Silvana Morello^1^, Maria Elena Capone^2^, Claudia Sorrentino^1,3^, Diana Giannarelli^4^, Gabriele Madonna^2^, Domenico Mallardo^2^, Antonio M Grimaldi^2^, Aldo Pinto^1,#^ and Paolo Antonio Ascierto^2,#^

###### ^1^Department of Pharmacy, University of Salerno, Fisciano, Italy; ^2^Melanoma, Cancer Immunotherapy and Innovative Therapies O.U., National Cancer Institute “G. Pascale”, Naples, Italy; ^3^PhD Program in Drug Discovery and Development, University of Salerno, Fisciano, Italy; ^4^Regina Elena National Cancer Institute, Rome, Italy

^#^These authors equally contributed to this work

*Journal of Translational Medicine 2018*, **16 (Suppl 1):**O18

**Background:** Anti-PD1 agents are successfully used in therapy to treat patients with advanced melanoma. Here, we retrospectively analysed the CD73 enzyme activity in the peripheral blood of in patients with metastatic melanoma receiving nivolumab. CD73 is an ectonucleotidase able to generate adenosine from AMP. Adenosine in the tumor microenvironment is a potent immune-suppressive mediator, so that inhibition of CD73-generating enzyme or blockade of adenosine receptors is a promising therapeutic strategy to fight cancer.

**Materials and methods:** CD73 enzyme activity was retrospectively analysed in the plasma of patients before receiving nivolumab. Levels of CD73 enzyme activity was correlated with the survival and progression-free survival of the patients analysed in this study and a multivariate analysis was performed to evaluate the prognostic value of this factor.

**Results:** 70% of the patients analysed in this study presented detectable CD73 activity in the plasma. High basal levels of sCD73 enzyme activity in serum were significantly associated with poor overall survival and progression-free survival in melanoma patients. In multivariate analysis, levels of CD73 significantly impact on both, overall survival and progression-free survival. Interestingly, we found that low levels of CD73 in the peripheral blood determined before treatment, were significantly associated with disease control rate to nivolumab. Patients who do not respond to nivolumab therapy instead presented higher levels of CD73 enzyme activity in the blood.

**Conclusion:** Although our results need to be confirmed and validated, they suggest that the activity of CD73 in the peripheral blood of patients with metastatic melanoma might be useful as prognostic factor and potentially as predictor of response to nivolumab treatment. We also postulate that increased levels of CD73 may contribute to affect the response of immunotherapeutic agents in cancer patients.

**Consent to publish:** All the patients provided written informed consent.

#### O19 Expression of CD73 on CD8+/PD-1+ cells as a new possible biomarker for advanced melanoma patients treated with nivolumab

##### Federica Fratangelo^1^, Silvana Morello^2^, Gabriele Madonna^1^, Maria Elena Capone^1^, Domenico Mallardo^1^, Rosaria Falcone^1^, Antonio M. Grimaldi^1^, Ester Simeone^1^, Vito Vanella^1^, Diana Giannarelli^3^, Claudia Sorrentino^2^, Aldo Pinto^2^ and Paolo A. Ascierto^1^

###### ^1^S.C. Oncologia Clinica Sperimentale Melanoma Immunoterapia e Terapie Innovative-IRCCS-Fondazione G. Pascale, Naples, Italy; ^2^Dipartimento di Farmacia, Università di Salerno, Fisciano, Italy; ^3^Istituto Nazionale Tumori Regina Elena, Roma, Italy

*Journal of Translational Medicine 2018*, **16 (Suppl 1):**O19

**Background:** Anti-programmed death (PD)-1 monoclonal antibodies have changed the prognosis of metastatic melanoma, improving overall survival [1]. However, still a proportion of patients is unresponsive to these compounds, indicating the presence of other immunosuppressive mechanisms. Thus, the identification of reliable biomarkers to predict the response to checkpoint blockades is still an unmet need.

Recent findings showed a tumor-induced immunosuppressive pathway, in which the extracellular adenosine produced by tumor-derived enzyme CD73 (5′-ectonucleotidase) promotes tumor growth by inhibiting immunosuppressive T-cell action [2]. Targeting adenosine generation by blockade of CD73 significantly enhances anti-tumor activity of anti-PD-1 drugs, inducing full regression in some tumor models [3].

The aim of the study was to investigate whether baseline levels of CD73+ on circulating CD8+, CD4+ and MDSCs cells could be considered as potential biomarkers in stage IV melanoma patients treated with nivolumab.

**Materials and methods:** Blood samples from 36 advanced melanoma patients were taken before nivolumab treatment; blood populations were measured in frozen peripheral blood mononuclear cells (PBMCs) that were thawed and then rested briefly, and subjected to flow cytometry analysis for myeloid-derived suppressor cells (MDSCs: CD14+ CD33+ CD11b+ HLA-DR-/low), CD8+ and CD4+, alone or in association with PD-1 and CD73+.

**Results:** Our data demonstrated that patients with lower baseline values of CD8+/PD-1+/CD73+ had high OS and PFS (34.8 and 19.2 months, respectively); conversely, patients with higher baseline frequency of these cells experienced lower OS and PFS (9.5 and 2.8 months, respectively; OS p < 0.003, PFS p < 0.007) (Tables 1, 2). In addition, increasing number of total CD8+ cells (p < 0.05) and especially of CD8+/PD-1+ cells (p < 0.04) were negatively correlated with survival (Table 1).

**Table 1:** Median OS (months) for patients with values under and over the median of CD8+, CD8+/PD-1+ and CD8+/PD-1+/CD73+

Factors (median)	Median OS (months) for patients with values under the median	Median OS (months) for patients with values over the median	p value
CD8+ (25.0)	20.4	6.9	0.05
CD8+/PD-1+ (7.8)	34.8	9.9	0.04
CD8+/PD-1+/CD73+ (2.3)	34.8	9.5	0.003

**Table 2:** Median PFS (months) for patients with values under and over the median of CD8+/PD-1+/CD73+

Factors (median)	Median PFS (months) for patients with values under the median	Median PFS (months) for patients with values over the median	p value
CD8+/PD-1+/CD73+ (2.3)	19.2	2.8	0.007

Furthermore, the baseline values of MDSCs/CD73^+^ and CD4+/CD73+ cells showed no significant differences in survival.

**Conclusions:** Our work indicates that the analysis of CD8+/PD-1+/CD73+ baseline levels in advanced melanoma patients treated with nivolumab could be associate to treatment outcome. Also, these preliminary results strengthen the therapeutic potential of anti-CD73 inhibitors, which are still in phase I of clinical trials, increasing the development of new treatment combinations strategies with other immune checkpoint monoclonal antibodies. Further studies on a larger number of patients are ongoing to confirm the data obtained.

**References**

1. Topalian SL, et al. Survival, durable tumor remission, and long-term safety in patients with advanced melanoma receiving nivolumab. J Clin Oncol. 2014;32:1020–30.
2. Ohta A. A metabolic immune checkpoint: adenosine in tumor microenvironment. Front Immunol. 2016;29(7):109.
3. Beavis PA, et al. CD73: a potential biomarker for anti-PD-1 therapy. Oncoimmunology 2015; 4(11):e1046675.

### Combination strategy session

#### O20 Prognostic impact of baseline tumor immune infiltrate on disease-free survival (DFS) in patients with completely resected, *BRAF*^V600^ mutation–positive (*BRAF*^V600+^) melanoma receiving adjuvant vemurafenib

##### Dirk Schadendorf^1^, Karl Lewis^2^, Michele Maio^3^, Lev Demidov^4^, Mario Mandalà^5^, Igor Bondarenko^6^, Paolo A. Ascierto^7^, Christopher Herbert^8^, Andrzej Mackiewicz^9^, Piotr Rutkowski^10^, Alexander Guminski^11^, Grant Goodman^12^, Brian Simmons^12^, Chenglin Ye^12^, Gregory Hooper^13^, Matthew J. Wongchenko^12^, Yibing Yan^12^

###### ^1^Department of Dermatology, University Hospital Essen, Essen, Germany; German Cancer Consortium, Heidelberg, Germany; ^2^University of Colorado Comprehensive Cancer Center, Aurora, CO, USA; ^3^Medical Oncology and Immunotherapy, Center for Immuno-Oncology, University Hospital of Siena, Siena, Italy; ^4^N N Blokhin Russian Cancer Research Center, Ministry of Health, Moscow, Russia; ^5^Department of Oncology and Haematology, Papa Giovanni XXIII Cancer Center Hospital, Bergamo, Italy; ^6^Dnipropetrovsk State Medical Academy, Dnipropetrovsk, Ukraine; ^7^Melanoma Unit, Cancer Immunotherapy and Innovative Therapies, Istituto Nazionale Tumori Fondazione “G. Pascale”, Naples, Italy; ^8^Bristol Haematology and Oncology Centre, Bristol, UK; ^9^Department of Cancer Immunology, Poznan University for Medical Sciences, Med-POLONIA, Poznan, Poland; ^10^Department of Soft Tissue/Bone Sarcoma and Melanoma, Maria Sklodowska-Curie Institute–Oncology Center, Warsaw, Poland; ^11^Melanoma Translational Research Group, Melanoma Institute Australia, Wollstonecraft, NSW, Australia; ^12^Genentech, Inc., South San Francisco, CA, USA; ^13^Roche Products Limited, Welwyn Garden City, UK

*Journal of Translational Medicine 2018*, **16 (Suppl 1):**O20

**Background:** The BRIM8 study (clinicaltrials.gov NCT01667419) evaluated the efficacy of adjuvant vemurafenib in patients with fully resected *BRAF*^V600+^ melanoma at high risk of recurrence.

**Materials and methods:** This 2-cohort (C) study randomized 498 adults with resected stage IIC, IIIA, or IIIB melanoma (C1) or stage IIIC melanoma (C2) 1:1 to vemurafenib 960 mg BID or placebo for 52 weeks. The primary endpoint was DFS. Hierarchical analysis of C2 before C1 was prespecified. The association of tumor immune infiltrate with DFS was explored retrospectively.

**Results:** In C2, median DFS was greater with adjuvant vemurafenib vs placebo (23.1 vs 15.4 mos), but the risk reduction was not statistically significant (log-rank *p* = 0.2598). Vemurafenib reduced DFS risk by 46% vs placebo in C1 (median not estimable vs 36.9 mos; log-rank *p* = 0.0010, not statistically significant as the primary DFS endpoint was not met in C2). Tumor samples were available for ≈60% of patients. In the pooled biomarker population, placebo-treated patients with < 1% CD8+ T cells in the tumor center had shorter median DFS vs patients with ≥ 1% CD8+ T cells (7.7 vs 47.8 months). The DFS benefit from vemurafenib vs placebo was greater in patients with < 1% CD8+ T cells in the tumor center (hazard ratio [HR] 0.56, 95% CI 0.34–0.92) than in patients with ≥ 1% CD8+ T cells (HR 0.77, 95% CI 0.48–1.22). Likewise, placebo-treated patients with < 5% PD-L1+ immune cells (IC) had shorter median DFS vs patients with ≥ 5% PDL1+ IC (7.2 vs 47.8 months). The DFS benefit from vemurafenib vs placebo was greater in patients with < 5% PD-L1+ IC in their tumors (HR 0.36, 95% CI 0.24–0.56) than in patients with ≥ 5% PD-L1+ IC (HR 0.99, 95% CI 0.58–1.69). The presence of CD8+ T cells and PD-L1+ IC are favorable prognostic factors for DFS.

**Conclusion:** Treatment with adjuvant vemurafenib may overcome the poor DFS prognosis associated with low CD8/PD-L1 expression in the tumor.

#### O21 4SC-202 plus anti-PD1: breaking PD1-refractoriness to increase efficacy of checkpoint inhibition in patients with advanced melanoma

##### Frank Hermann, Roland Baumgartner, Astrid Ammendola, SvetlanaHamm, René Bartz

###### 4SC AG, Martinsried; Germany

*Journal of Translational Medicine 2018*, **16 (Suppl 1):**O21

**Background:** Despite successes in the treatment of melanoma patients with checkpoint inhibitors (anti-PD1 antibodies), the majority of patients do not respond to checkpoint inhibition alone and a high unmet medical need remains for these patients. One promising approach is to increase the number of patients benefiting from checkpoint inhibition by enhancing the immunogenicity and alter the tumor microenvironment from a more immune-deserted to an immune-inflamed phenotype by means of combination therapy. Epigenetic modulation has been reported as one key determining factor in shaping the immune microenvironment and compounds altering these processes [e.g. histone deacetylases (HDAC) inhibitors] are particularly promising.

**Results:** Here, we report results for 4SC-202, an orally available clinical stage HDAC inhibitor, and outline the further clinical development. 4SC-202 treatment led to an increase of MHC class II molecules and enhanced expression of inflammatory markers like IFN-γ and various chemokines in tumors. Furthermore, detailed analysis of the tumor microenvironment in tumor bearing animals revealed that 4SC-202 strongly altered the immune cell composition and particularly the number of cytotoxic T cells (CTL) was markedly increased. Importantly, subsequent combination treatment of 4SC-202 with checkpoint inhibitors in syngenic animal models showed a strong synergistic effect resulting in an increased tumor growth reduction.

For the further clinical development, start of a Phase Ib/II clinical study (‘SENSITIZE’; ClinicalTrials.gov Identifier: NCT0327866) is planned by the end of this year. This study, conducted in Germany with up to 6 sites (~ 30 patients) will enroll patients with advanced cutaneous melanoma who are refractory/non-responding to treatment with anti-PD-1 antibodies. These patients clearly represent a population with a high unmet medical need and might be characterized by an unfavorable tumor immunology and microenvironment for immunotherapy in general and checkpoint inhibition in particular.

**Conclusion:** We hypothesize that addition of 4SC-202 to anti-PD-1 antibody treatment may lead to increased immunogenicity of the tumor, an inflamed tumor microenvironment and ultimately to clinical benefit in anti-PD-1 refractory/non-responding advanced melanoma patients.

## Melanoma bridge 2017—poster presentations

### P4 Insights into the patient perspective: uncovering the clinical experiences and unmet needs for melanoma patients

#### Bulotta Alessandra^1^, Colombo Letizia^1^, Mirabile Aurora^1^, Lazzari Chiara^1^, Mercuri Santo Raffaele^2^, Parolini Danilo^4^, Rizzo Nathalie^5^, Martella Stefano^3^, Modorati Giulio^6^, Brianti Pina^2^, Cestone Enza^2^, Bellinzona Federica^2^, Miserocchi Elisabetta^6^, Gianni Luca^1^ and Gregorc Vanesa^1^

##### ^1^Department of Oncology, Division of Experimental Medicine, San Raffaele Scientific Institute, Milan, Italy; ^2^Dermatology and Cosmetology Unit-San Raffaele Scientific Institute, Milan, Italy; ^3^Division of Plastic and Reconstructive Surgery, San Raffaele Scientific Institute, Milan, Italy; ^4^Unit of Gastrointestinal Surgery, San Raffaele Scientific Institute, Milan, Italy; ^5^Department of Pathology, San Raffaele Scientific Institute, Milan, Italy; ^6^Department of Ophthalmology, San Raffaele Scientific Institute, Milano, Italy

*Journal of Translational Medicine 2018*, **16 (Suppl 1):**P4

**Background:** Data from the literature indicate that the perception of patients with melanoma regarding the support received from healthcare services is generally inadequate with respect to the patients’ need. Approximately 30% of melanoma patients face significant physical and psychological issues related to the diagnosis, treatment and follow-up [1-3]. The aim of this study is to evaluate the patients’ specific clinical and communication needs in order to maximize their quality of health care.

**Materials and methods:** A predefined questionnaire was submitted to 130 patients with new diagnoses of melanoma (stage I-IV), followed at San Raffaele Hospital (HSR) from January 2015 to January 2017. The study was approved by the Ethical Committee. The participants were recruited using non-probability purposing sampling techniques. The questionnaires were administered by email or telephone. It included 34 items, evaluating patients’ demographics, education and employment status, basic knowledge on melanoma, and the understanding of diagnosis, stage, treatment details, prognosis, screening behaviors, quality of medical-patient communication and the perception for healthcare personnel support. The questionnaires were adapted from those used in Australia and New Zealand [4–5]. The reliability of the instrument was tested using test–retest reliability in IBM SPSS version 18. Statistical analysis was carried out using excel and PASW version 18 (SPPSS, Inc, II), adjusted by age, gender, education and stage of tumor.

**Results:** One hundred and thirty patients accessed the questionnaire. Fifty-two have been already completed. Here we present preliminary date. In Table 1 patients’ characteristics.

**Table 1:** Patients’ characteristics

Patients’ characteristics	Total no 52 (100%)
Gender	
Female	33 (63.5%)
Male	19 (36.5%)
Education level	
< high school	8 (15.4%)
High school	8 (15.4%)
Apprenticeship training	21 (40.4%)
Bachelors/masters	3 (5.8%)
Doctorate	12 (23.1%)
Tumour’ sites	
Torso	25 (48.1%)
Limbs	19 (36.5%)
Head or neck	5 (9.6%)
Others	3 (5.8%)
Tumour’ stage	
Stage I	29 (55.8%)
Stage II	4 (7.7%)
Stage III	3 (5.8%)
Stage IV	4 (7.7%)
Unknown to the patient	12 (23.1%)

Patients were grouped according to age (< 50, 50–70 and > 70 years) and the level of education (high school or less and vocational training). Sixty-three per cent of patients were satisfied about the support received, and 51.4% received enough information regarding diagnosis, prevention, treatment options and recurrence of the disease. Patients underline that like to have a single healthcare consultant.

**Conclusions:** This is a deepened patient-centred approach conceived for melanoma patients that would provide a perspective on melanoma care, highlighting the areas that require the definition of a new protocol based on patients and their caregivers’ needs. In Table 1 patients’ characteristics.

**References**

1. Living as a melanoma skin cancer survivor.” American Cancer Society, www.cancer.org/cancer/melanoma-skin-cancer/after-treatment/follow-up.html.
2. Chiesi, Francesca, et al. Assessing unmet needs in patients with cancer: an investigation of differential item functioning of the needs evaluation questionnaire across gender, age and phase of the disease. PLOS ONE. Public Library of Science, 25th July 2017.
3. Sanson-Fisher, R, et al. The unmet supportive care needs of patients with cancer. supportive care review group. Cancer. US National Library of Medicine 1 Jan. 2000.
4. Mitchell, Janine, et al. The experience of melanoma follow-up care: an online survey of patients in Australia. J Skin Cancer. Hindawi Publishing Corporation. 2014. www.ncbi.nlm.nih.gov/pmc/articles/PMC4254069/.
5. Kasparian, N A. Psychological stress and melanoma: are we meeting our patients’ psychological needs?” Clinics in Dermatology. US National Library of Medicine. www.ncbi.nlm.nih.gov/pubmed/23245972.

#### P5 Oncolytic immunotherapy with PV-10

##### Sanjiv Agarwala, B. Mark Smithers, Axel Haushild, Paolo A. Ascierto, Eric Watcher

###### ^1^St Luke’s University Hospital and Health Network, Easton, Pennsylvania, USA; ^2^Princess Alexandra Hospital, Brisbane, Queensland, Australia; ^3^Department of Dermatology, University Hospital Schleswig–Holstein, Kiel, Germany; ^4^Istituto Nazionale Tumori IRCCS Fondazione “G Pascale”, Naples, Italy; ^5^Provectus Biopharmaceuticals, Inc., Knoxville, Tennessee, United States

*Journal of Translational Medicine 2018*, **16 (Suppl 1):**P5

**Background:** PV-10 is an investigational small molecule oncolytic immunotherapy (10% w/v rose bengal disodium for injection). Intralesional (IL) injection can elicit a primary, local effect in injected tumor tissue (immunogenic cell death and release of tumor antigens) capable of stimulating a secondary, functional systemic immune response (including tumor-specific reactivity in circulating T cells in treatment-refractory patients). PV-10 has been investigated in Phase 1 and 2 clinical trials in 130 patients with melanoma; an expanded access protocol accrued an additional 180 melanoma patients.

**Materials and methods:** PV-10 is currently under clinical investigation in two melanoma studies. Phase 3 study PV-10-MM-31 (NCT02288897), an international multicenter, randomized controlled trial (RCT), is assessing effectiveness of PV-10 compared to the investigator’s choice of systemic chemotherapy or intralesional oncolytic viral therapy in up to 225 patients with locally advanced cutaneous melanoma (AJCC Stage IIIB to IV M1a). The primary endpoint is progression-free survival (PFS) assessed via RECIST 1.1. Phase 1b/2 combination study PV-10-MM-1201 (NCT02557321), an international multicenter, open-label, sequential phase study, is assessing safety and efficacy of PV-10 in combination with systemic immune checkpoint inhibition. Stage IV metastatic melanoma patients with at least one injectable cutaneous or subcutaneous lesion who are candidates for pembrolizumab are eligible for study participation. In the Phase 1b portion of the study, up to 24 subjects will receive the combination (i.e., PV-10 + SoC) with PFS and objective response (OR) as key secondary endpoints. In the Phase 2 portion of the study up to 120 study participants will be randomized 1:1 to receive either the combination or pembrolizumab alone (i.e., PV-10 + SoC vs SoC) with PFS as the primary endpoint.

**Results:** Both studies are currently enrolling patients. The Phase 3 study is open at centers in the USA, AUS, Italy and Germany; additional regulatory approval has been received in Mexico, with approval pending in France, Poland and Argentina. The Phase 1b study is open at centers in the USA and AUS. To date, no unexpected safety signals have been observed in either study, and Phase 2 of the combination study is expected to begin in early 2018. Preliminary efficacy data from Phase 1b of the combination trial-in-progress will be presented.

**Conclusion:** Pivotal testing of single agent PV-10 is ongoing. Conclusion of Phase 1b testing of PV-10 in combination with checkpoint inhibition is anticipated to lead to Phase 2 randomized testing in 2018.

#### P6 The reciprocal interplay between miR-579-3p and MITF controls melanoma proliferation and resistance to target therapy

##### Luigi Fattore^1^, Ciro Francesco Ruggiero^2^, Domenico Liguoro^3^, Andrea Cerri^4^, Maria Elena Pisanu^3^, Paolo Antonio Ascierto^1^, Rita Mancini^3^, Gennaro Ciliberto^4^

###### ^1^Istituto Nazionale per lo Studio e la Cura dei Tumori “Fondazione G. Pascale”, Naples, Italy; ^2^Dipartimento di Medicina Sperimentale e Clinica, Università degli Studi di Catanzaro “Magna Graecia”, Catanzaro, Italy; ^3^Dipartimento di Medicina Clinica e Molecolare, Sapienza Università di Roma, Rome, Italy; ^4^Regina Elena National Cancer Institute, Rome, Italy

*Journal of Translational Medicine 2018*, **16 (Suppl 1):**P6

**Background:** MAPK signaling is the main oncogenic driver in metastatic melanomas bearing mutations in BRAF kinase. These tumors are currently treated with the combination of BRAF/MEK inhibitors (MAPKi), but this therapy is plagued by drug resistance. In this context we recently focused on non-mutational mechanisms contributing to the development of drug resistance and in particular on the role of miRNAs. This led us to the discovery of miR-579-3p as an antagonist of melanoma progression and resistance. miR-579-3p targets both BRAF and MDM2 oncogenes and its expression is strongly downregulated during melanoma development and during progression to drug resistance. In the present work, we studied the mechanisms regulating the expression of miR-579-3p and the interplay with MITF, the master regulator of the melanocyte lineage.

**Materials and methods:** JASPAR open database was queried to identify potential transcription factors binding to the miR-579-3p promoter, which were confirmed through silencing, luciferase and Chromatin-Immunoprecipation experiments. Three melanoma cell lines (M14, LOX IMVI and WM266) bearing different mutations in the BRAF oncogene were exposed to MAPKi for different times and then collected for protein and RNA extraction. FFPE samples from matched tumors before and after the development of resistance to MAPKi were used for total RNA extraction and analysis through qRT-PCR.

**Results:** miR-579 gene is located within intron 11 of the Zinc Finger Recombinase (ZFR) gene. Bioinformatic analysis allowed us to identify two putative MITF binding sites within ZFR gene promoter. Gene silencing, luciferase assays and ChIP experiments confirmed that MITF binds to the ZFR promoter and that acts a positive regulator of miR-579-3p transcription. Moreover, miR-579-3p by targeting BRAF is able to stabilize MITF protein thus inducing its own transcription in a positive feedback regulatory loop. In line with these findings, BRAF mutated melanoma cells exposed to MAPK inhibitors undergo a rapid upregulation of both MITF and miR-579-3p which corresponds to cell growth inhibition. On the opposite drug resistant melanoma cells show downregulation of MITF and miR-579-3p in parallel. Simultaneous loss of both MITF and miR-579-3p is also observed in tumor samples from patients after development of drug resistance.

**Conclusions:** In this work we uncover a positive feedback regulatory loop involving MITF and the on co-suppressor miRNA miR-579-3p which inhibits melanoma proliferation. Drug resistant melanoma cells escape this mechanism by downregulating both MITF and miR-579-3p. We are currently investigating the molecular basis of the “switch” from MITF^+^/miR-579-3p^+^ drug sensitive to the MITF^low^/miR-579-3p^low^ drug insensitive status.

